# CpG signalling, H2A.Z/H3 acetylation and microRNA-mediated deferred self-attenuation orchestrate foetal NOS3 expression

**DOI:** 10.1186/s13148-014-0042-4

**Published:** 2015-02-08

**Authors:** Jan Postberg, Miriam Kanders, Sakeh Forcob, Rhea Willems, Valerie Orth, Kai Oliver Hensel, Patrick Philipp Weil, Stefan Wirth, Andreas Christoph Jenke

**Affiliations:** HELIOS Childrens Hospital, Centre for Biomedical Education and Research, Witten/Herdecke University, Wuppertal, Germany; Children’s Hospital, Helios Klinikum Wuppertal, 42283 Wuppertal, Germany

**Keywords:** Placental perfusion, miRNA, Nitric oxide, Intrauterine growth retardation

## Abstract

**Background:**

An adverse intrauterine environment leads to permanent physiological changes including vascular tone regulation, potentially influencing the risk for adult vascular diseases. We therefore aimed to monitor responsive NOS3 expression in human umbilical artery endothelial cells (HUAEC) and to study the underlying epigenetic signatures involved in its regulation.

**Results:**

NOS3 and STAT3 mRNA levels were elevated in HUAEC of patients who suffered from placental insufficiency. 5-hydroxymethylcytosine, H3K9ac and Histone 2A (H2A).Zac at the NOS3 transcription start site directly correlated with NOS3 mRNA levels. Concomitantly, we observed entangled histone acetylation patterns and NOS3 response upon hypoxic conditions *in vitro*. Knock-down of either NOS3 or STAT3 by RNAi provided evidence for a functional NOS3/STAT3 relationship. Moreover, we recognized massive turnover of Stat3 at a discrete binding site in the NOS3 promoter. Interestingly, induced hyperacetylation resulted in short-termed increase of NOS3 mRNA followed by deferred decrease indicating that NOS3 expression could become self-attenuated by co-expressed intronic 27 nt-ncRNA. Reporter assay results and phylogenetic analyses enabled us to propose a novel model for STAT3-3′-UTR targeting by this 27-nt-ncRNA.

**Conclusions:**

An adverse intrauterine environment leads to adaptive changes of NOS3 expression. Apparently, a rapid NOS3 self-limiting response upon ectopic triggers co-exists with longer termed expression changes in response to placental insufficiency involving differential epigenetic signatures. Their persistence might contribute to impaired vascular endothelial response and consequently increase the risk of cardiovascular disease later in life.

**Electronic supplementary material:**

The online version of this article (doi:10.1186/s13148-014-0042-4) contains supplementary material, which is available to authorized users.

## Background

Epidemiological evidence suggests that early environmental factors such as placental insufficiency correlate with increased disease risks later in life, e.g. cardiovascular disease [1]. The phenotypic changes conveying these risks are necessarily due to deregulated expression of specific genes during the acute phase of the insult. Gene expression changes are known to be strongly associated with the plasticity of chromatin, whose structure can be influenced by CG dinucleotide (CpG) signalling and post-translational modifications (PTMs) of histones. Their combinatorial signatures control the spatiotemporal expression of genes in a potentially heritable way. In more detail, desoxyribonucleic acid (DNA) methylation targets cytosines (5-methylcytosine (5meC)) predominantly at isolated asymmetric CpG motifs (‘open sea’) or in the genomic context of so-called ‘CpG islands’ and adjacent regions (‘shores/shelves’) [2-4]. 5meC enrichment in promoters is frequently associated with transcriptional repression [5]. Recently, hydroxymethylated cytosines (5-hydroxymethylcytosine (5hmeC)) were recognized as another functional DNA modification. Currently, the most widely accepted hypothesis is that 5hmeC is an intermediate state of active DNA demethylation conferred by members of the Tet protein family [6] and thus influencing gene expression [7,8]. Yet, another level of gene expression regulation is conveyed by PTMs of histones, which occur in a dynamic fashion through variable combinations at a given site. PTMs can influence chromatin compaction either directly or in conjunction with ‘reader’ proteins, thus regulating either activation or repression of genes [9].

The placenta represents the interface between the foetal and maternal organism. Its perfusion is therefore closely linked to foetal well-being and critically influences foetal development [10]. One important factor influencing short- and long-term placental perfusion is hypoxia [11-13], representing the most adverse intrauterine *milieu* for the foetus. In response—particularly in chronic hypoxia [14]—foetal circulation compensatory mechanisms involve nitric oxide (NO) synthesis by endothelial nitric oxide synthase (eNOS), which eventually contributes to blood vessel tone regulation. NOS3 mRNA synthesis, which gives rise to the eNOS protein, was recently found to be diminished in response to hypoxia in human umbilical vein endothelial cells (HUVEC) [15]. In contrast, NOS3 mRNA was increased in human umbilical artery endothelial cells (HUAEC) [16]. On the molecular level, NOS3 expression and eNOS activation are complex, mainly mediated by PI3K/Akt- or AC/PKA-signalling involving transcriptional, post-transcriptional and post-translational factors [17,18]. Attributes of current short-term regulation concepts involve rapid modulation of NOS3 mRNA stability or activation of the eNOS protein via serine phosphorylation [19]. Adaptations to long-term stimuli (e.g. chronic hypoxia) apparently involve regulation on the transcriptional level and hence epigenetic plasticity [20]. Recently, it has been shown in cell culture experiments that hypoxic repression of eNOS transcription in HUVEC is coupled with eviction of promoter histones [21]. Further detailed insights into this aspect of NOS3/eNOS regulation are lacking to date, particularly in relation to its corresponding clinical significance.

We therefore undertook comprehensive analyses on the regulation of NOS3 expression in foetal HUAEC cells. This included NOS3 mRNA quantification, genotyping of an intronic putative non-coding RNA (ncRNA) locus suspicious for pathomechanistic relevance in cardiovascular diseases [22], monitoring of CpG signalling at the NOS3 promoter and quantification of PTMs relevant for chromatin structure regulation. We further tested a yet sparsely evidenced connection between Stat3 signalling and NOS3/eNOS regulation [23].

## Results

### Impaired placental perfusion is associated with enriched NOS3 mRNA independent of tandem repeat polymorphisms of the NOS3 intronic 27 nt-ncRNA

**Figure 1:**
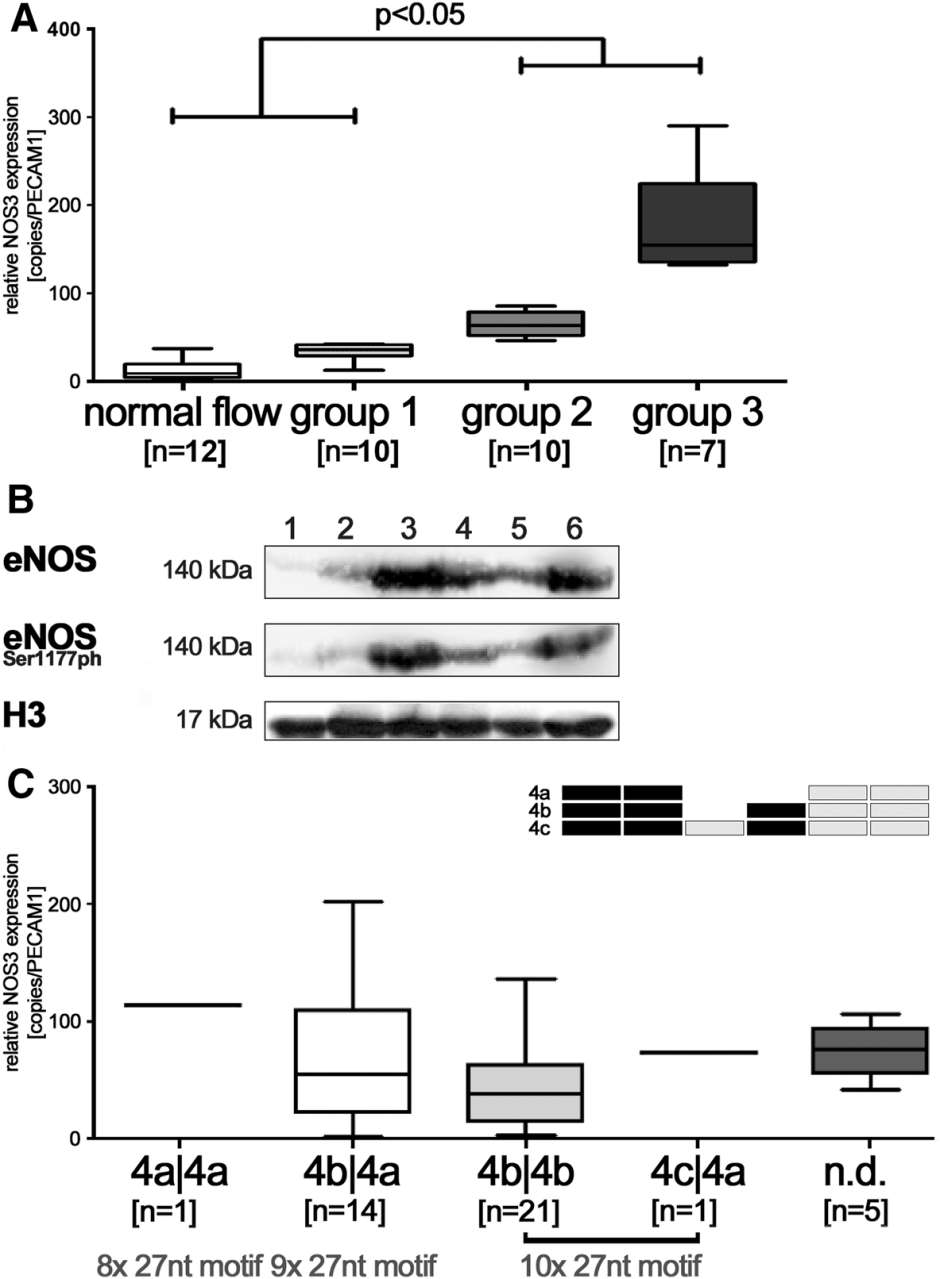
**NOS3 mRNA correlates with placental perfusion indices and does not depend on NOS3 intronic 27 nt-ncRNA polymorphism. (A)** NOS3 mRNA levels increased >15-fold in patients suffering from severe impairment of placental perfusion (group 3). **(B)** Protein levels of eNOS and eNOS(Ser1177ph) correlated with NOS3 mRNA levels. NOS3 mRNA levels were as follows: 12.9 (lane 1), 22.6 (lane 2), 157.7 (lane 3), 56.8 (lane 4), 74.7 (lane 5), 136.9 (lane 6). **(C)** No correlation between NOS3 mRNA levels and genotype at the 27 nt-ncRNA locus in intron 5 of the NOS3 gene was found. In human alleles, two tandem-repeated homologous motifs were found in typical allele-specific order (top right). The number of patients analysed for each group is stated (*n*).

Thirty-nine infants were included in this study. Placental and foetal perfusion indices were assessed from all patients within 24 h prior to delivery and classified to have either normal flow patterns (group 0; *n* = 10), mild (group 1; *n* = 12), moderate (group 2; *n* = 10) or severe impairment (group 3; *n* = 7) (Table 1). Epidemiological parameters such as maternal age, parity, birth weight, gestational age (GA), pH and Apgar at 5 min did not differ between the groups (except from the ponderal index) (Table 1). From all patients, HUAEC could be isolated to obtain sufficient amounts of ribonucleic acid (RNA) for cDNA synthesis and gDNA for genotyping. Amounts of gDNA were suitable from 13 patients to enable (h)methylated DNA immunoprecipitation (MeDIP). Quantitative real-time PCR (qPCR) revealed that NOS3 mRNA levels differed significantly between the flow groups, whereas NOS2 mRNA levels, encoding inducible nitric oxide synthase (iNOS), did not vary (data not shown). Lowest NOS3 mRNA levels were observed in patients with normal placental perfusion (group 0, median 13.5 copies per PECAM1, 2.8–37.6) and highest in patients with severely impaired placental perfusion (group 3, median 218.7 copies per PECAM, 107–386) (Figure 1A). In all cases, levels also correlated with total cellular eNOS and eNOS(Ser1177ph) protein levels (Figure 1B). Since copy number polymorphisms of a 27-bp tandem repeat (variable number tandem repeat (VNTR)) in intron 5 had previously been associated with adult cardiovascular disease [24], we determined this VNTR and its zygosity for all patients. No correlation between different 27-bp VNTR genotypes and NOS3 mRNA levels or placental perfusion was observed (Figure 1C).

**Table 1:** **Epidemiological characteristics**

	**Total (** ***n*** **= 39)**	**Flow group 0 (** ***n*** **= 10)**	**Flow group 1 (** ***n*** **= 12)**	**Flow group 2 (** ***n*** **= 10)**	**Flow group 3 (** ***n*** **= 7)**
Gestational age (SD)	31.9 ± 4.9	31.8 ± 4.8	31.6 ± 5.7	32.1 ± 4.9	31.2 ± 4.6
Birth weight in g (SD)	1,655 ± 917	1,746 ± 1,008	1,652 ± 810	1,636 ± 909	1,336 ± 657
Birth length in cm (SD)	41.4 ± 6.1	41.0 ± 7.1	40.7 ± 6.1	41.9 ± 5.5	40.7 ± 5.2
Maternal age (SD)	31.9 ± 4.9	31.8 ± 4.8	31.6 ± 5.7	31.2 ± 4.9	31.2 ± 4.6
Ponderal index	21.9 ± 3.5	23.3 ± 2.8	22.9 ± 3.5	19.7 ± 3.0	18.8 ± 1.98
Apgar at 5 min	8.5 ± 1.2	8.5 ± 1.4	8.7 ± 1.3	8.4 ± 1.3	8.3 ± 1.1
pH at birth	7.34 ± 1.1	7.34 ± 1.2	7.33 ± 1.1	7.35 ± 1.4	7.32 ± 1.2
Female (%)	54	42	58	60	57
HELLP syndrome (%)	7.7	-	-	10	28.6
Preeclampsia (%)	15.4	-	8.3	20	42.9

### High-5-hydroxymethylcytosine levels adjacent to the NOS3 transcription start site (TSS) correlate with impaired placental perfusion and enriched NOS3 mRNA

**Figure 2:**
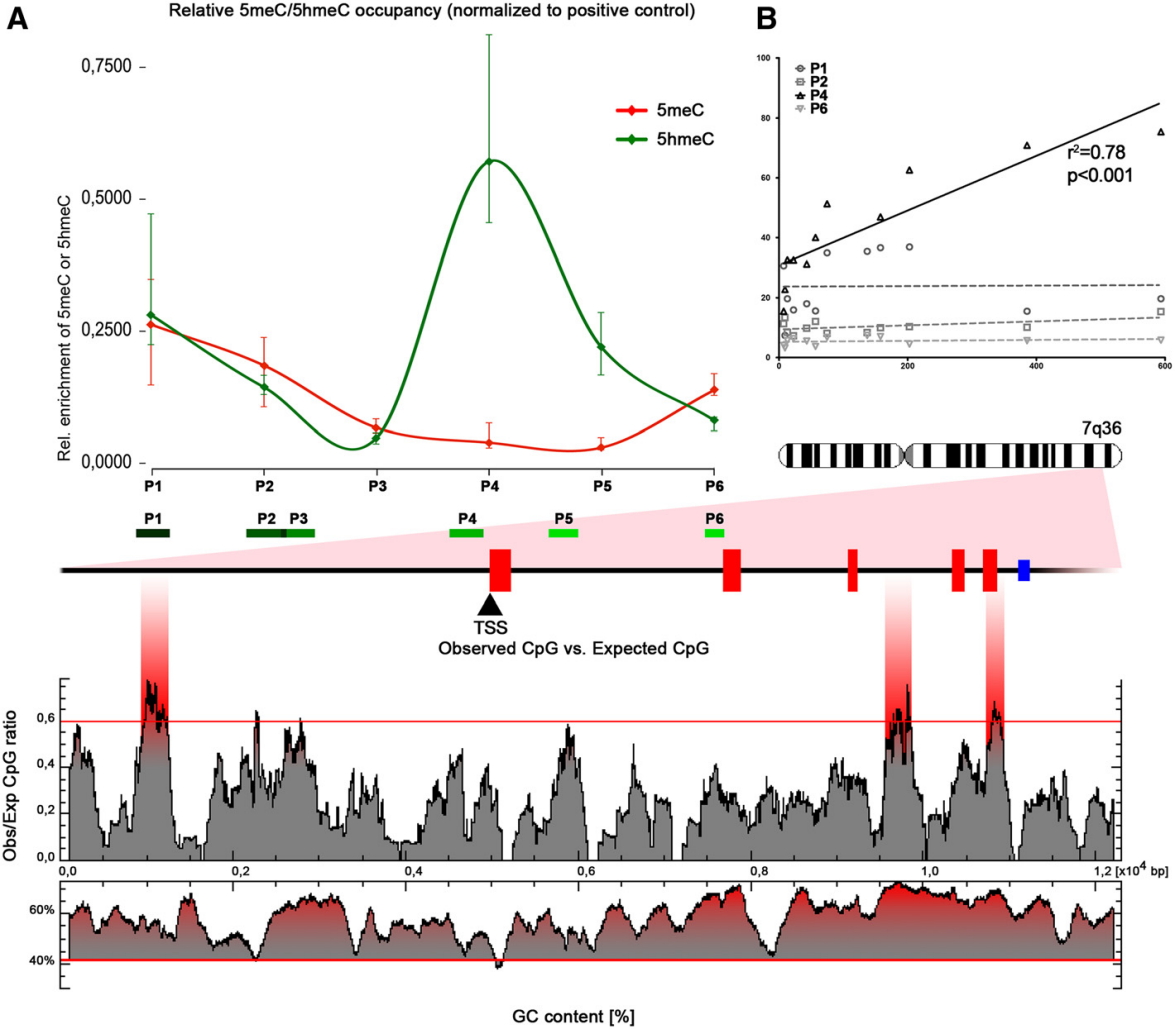
**5-hydroxymethylcytosine (5hmeC) levels adjacent to the NOS3 TSS correlate with impaired placental perfusion and upregulation of NOS3 mRNA. (A)** 5meC (red line) within the NOS3 promoter exhibited a local depression in all patients with minimal variations. In contrast, the amounts of precipitated 5hmeC-enriched DNA (green line) showed a local peak, particularly at the TSS (P4) with significant variations between individuals. In the gene structure cartoon (below), the first 5 NOS3 exons are marked as red boxes and a black arrow indicates the TSS. CpG-enriched regions analysed by qPCR are shown as green boxes (P1 to P6). The blue box marks the location of the 27 nt-ncRNA in intron 5. **(B)** 5hmeC levels at the TSS (P4), but not at other CpG-rich loci, directly correlate with NOS3 mRNA levels.

To understand the regulation of differential NOS3 expression in response to changes in placental perfusion, we compared epigenetic biomarkers at the levels of DNA CpG signalling between patients with normal and impaired placental flow indices. Initially, we analysed a 12-kb sequence fragment of the human NOS3 gene (−5 kb upstream of transcription start site (TSS) and +7 kb downstream of TSS) for putative target sites of differential 5meC/5hmeC signatures (Figure 2A) followed by (h)MeDIP and qPCR analyses to discover whether these putative target sites were pulled down by antibodies specific for either 5meC or 5hmeC (Table 2, Figure 2A). Importantly, both antibodies turned out to be highly specific with a recovery rate of a total methylated control fragment from input DNA exceeding 45% using the anti-5meC mAb, whereas it was below 2.5% for total hydroxymethylated control DNA and below 1% for unmodified control DNA. Using the anti-5hmeC mAb, recovery of total hydroxymethylated control DNA was greater than 69%, whereas it was close to 0.01% for total methylated or unmodified DNA (Additional file 1: Data 1). We observed considerable inter- and intraindividual differences in overall 5meC levels in the NOS3 promoter across the analysed 12-kb fragment (Additional file 1: Data 1), but no correlation between 5meC levels and NOS3 mRNA levels. Interestingly, regardless of interindividual differences, a common pattern with local 5meC depression at the TSS and elevated methylation at the boundaries was recognizable (Figure 2A). Importantly, the amount of 5meC associated with the above-mentioned depression was reminiscent of a hypomethylated locus within the glyceraldehyde-3-phosphate dehydrogenase (GAPDH) promoter. On the other hand, the increased levels of 5meC at both boundaries were reminiscent of a hypermethylated locus within the transcriptionally mostly silent testis-specific histone 2B (TSH2B) gene (Additional file 1: Data 1). To further analyse whether the local 5meC depression at the NOS3 TSS indicated a massive turnover of 5meC at this site, we decided to probe the same loci as described above for 5hmeC enrichment, since recent data suggested that 5hmeC represents an intermediate state of DNA demethylation by Tet protein family members [7]. Strikingly, we observed an almost inverse pattern of 5hmeC at the TSS when compared to 5meC. At this site, 5hmeC enrichment differed significantly between individual patients and exhibited direct correlation with the grade of impaired placental perfusion and elevated NOS3 mRNA levels (Figure 2A,B).

**Table 2:** **Oligonucleotides used in this study**

**Name**	**Sequence 5′ to 3′**	**Purpose**
NOS3_P1+	tcagcctgcccctgaaac	(h)MeDIP/ChIP-qPCR
NOS3_P1−	catccgtgggaacttgaaat	(h)MeDIP/ChIP-qPCR
NOS3_P2+	atttgtggggaaatcaaacg	(h)MeDIP/ChIP-qPCR
NOS3_P2−	cccttcttgagacagccaca	(h)MeDIP/ChIP-qPCR
NOS3_P3+	attgtgctgaaatcgctcct	(h)MeDIP/ChIP-qPCR
NOS3_P3−	agtaccccacccttgcacta	(h)MeDIP/ChIP-qPCR
NOS3_P4+	agaagaagggcctcacatca	(h)MeDIP/ChIP-qPCR
NOS3_P4−	tcctgagtcacctgttctgtg	(h)MeDIP/ChIP-qPCR
NOS3_P4a+	cacaagactccagggaagca	(h)MeDIP/ChIP-qPCR
NOS3_P4a−	ctgcagaaggtgctggtgg	(h)MeDIP/ChIP-qPCR
NOS3_P5+	ctggaatcccagcccatt	(h)MeDIP/ChIP-qPCR
NOS3_P5−	cctcccggagtgattctcat	(h)MeDIP/ChIP-qPCR
NOS3_P6+	gctcccacttatcagcctca	(h)MeDIP/ChIP-qPCR
NOS3_P6−	agccctggccttttccttag	(h)MeDIP/ChIP-qPCR
NOS3_P7+	gggggagatccttgcctttt	(h)MeDIP/ChIP-qPCR
NOS3_P7−	ccaggtcttcactactgggc	(h)MeDIP/ChIP-qPCR
NOS3_cDNA+	tccacatgagctggggtaggca	cDNA/qPCR
NOS3_cDNA−	gggactgtgccagatgttaggaga	cDNA/qPCR
STAT3+	gacattcccaaggaggaggc	cDNA/qPCR
STAT3−	ggtcttcaggtatggggcag	cDNA/qPCR
STAT3alpha+	caggtagcgctgccccatac	cDNA/qPCR
STAT3alpha−	atggtattgctgcaggtcgtt	cDNA/qPCR
STAT3beta+	gagccaggagcatcctgaag	cDNA/qPCR
STAT3beta−	tccaaactgcatcaatgaatggt	cDNA/qPCR
ATP6+	gccctagcccacttcttacc	HKG
ATP6−	ccagggctattggttgaatg	HKG
BACT+	gtcgacaacggctccggcatg	HKG
BACT−	atgtcgtcccagttggtgacg	HKG
GAPDH+	aggtcggagtcaacggattt	HKG
GAPDH−	tggaagatggtgatgggattt	HKG
PECAM1+	tgcctaacaccgcaagggcaa	HKG
PECAM1−	actctgtggaccccaggttgaga	HKG
RPL19+	atcgatcgccacatgtatca	HKG
RPL19−	ctggtcagccaggagctt	HKG
VIL1+	gcctcgatggaagcaacaaaacct	HKG
VIL1−	agggtactgctttacaaccacagc	HKG
NOS3_intron5out+	caggcacctaccagcttagg	Nested PCR/genotyping
NOS3_intron5out−	ggaactggggtagcagtgg	Nested PCR/genotyping
NOS3_intron5in+	ggatccagtgggggaagc	Nested PCR/genotyping
NOS3_intron5in−	ttctctcttgggggagaagc	Nested PCR/genotyping
NOS3_intron5_antisense+	aggctgctcctgctactgac	cDNA/qPCR
NOS3_intron5_antisense−	ctggaggaggggaaagaagt	cDNA/qPCR
siRNA_STAT3	AAGGAGGAGGCAUUCGGAAAGUAdTdT	siRNA
	dTdTUUCCUCCUCCGUAAGCCUUUCAU	
siRNA_NOS3	AAGAGUUAUAAGAUCCGCUUCdTdT	siRNA
	dTdTUUCUCAAUAUUCUAGGCGAAG	
Luci+	tgcaaaagatcctcaacgtg	cDNA/qPCR
Luci−	aatgggaagtcacgaaggtg	cDNA/qPCR
27 nt-siRNA motif 1	gaagucuagaccugcugcgggggugag	siRNA
	cuucagaucuggacgacgcccccacuc	
27 nt-siRNA motif 2	gaagucuagaccugcugcaggggugag	siRNA
	cuucagaucuggacgacguccccacuc	
NOS3-2800+	aaacaccccgcctcctaac	ChIP-qPCR
NOS3-2800−	tgcatgcacagtcacaca	ChIP-qPCR
NOS3-1554+	cagccgaacaccaaatctcc	ChIP-qPCR
NOS3-1554−	cagccctgccaagaatgatg	ChIP-qPCR
NOS3-1250+	ccctgtccagagagcattca	ChIP-qPCR
NOS3-1250−	ttgggtttgaattggggcag	ChIP-qPCR
NOS3-929+	cctctccattgcctccagag	ChIP-qPCR
NOS3-929−	gttcctctctgtggggatcc	ChIP-qPCR
NOS3-804+	cacacaggttcagagcacac	ChIP-qPCR
NOS3-804−	atgatctctgggtggctgtc	ChIP-qPCR
NOS3-620+	gatcatggagaaggggacgt	ChIP-qPCR
NOS3-620−	cattgcccgcagtattcctc	ChIP-qPCR

### Turnover of histone acetylation adjacent to the NOS3 TSS correlates with impaired placental perfusion and differential NOS3 expression

For transcriptional repression, 5meC presumably must be read out by 5meCpG-binding proteins, which entails assembly of repressive complexes. These complexes can contain histone deacetylase (HDAC) activity [5,25-27]. Vice versa, local chromatin conformation could also depend on 5hmeC signatures. To obtain comprehensive insight into the epigenetic regulation of NOS3 expression, we thus decided to analyse whether modifications of the chromatin signature might also be involved in NOS3 regulation. Out of seven patients’ samples belonging either to flow group 0 (*n* = 3) or flow group 3 (*n* = 4), we could purify sufficient amounts of chromatin for chromatin immunoprecipitation (ChIP) followed by qPCR analyses. We focused these analyses on histone modifications frequently associated with the promoters of either transcriptionally competent/active genes (histone 2A (H2A).Zac, H3K9ac) or repressed genes (H3K9me3). Further, we targeted histone 3 (H3) concomitantly modified at lysine-9 and serine-10 (H3K9me3/S10ph), since it has been reported that phosphorylation of serine-10 is sufficient to eject heterochromatin protein 1 (HP1) bound at H3K9me3 [28] thus counteracting heterochromatin formation. This selection enabled us to analyse three switch positions at one discrete PTM target site, which could potentially be associated with biological consequences.

**Figure 3:**
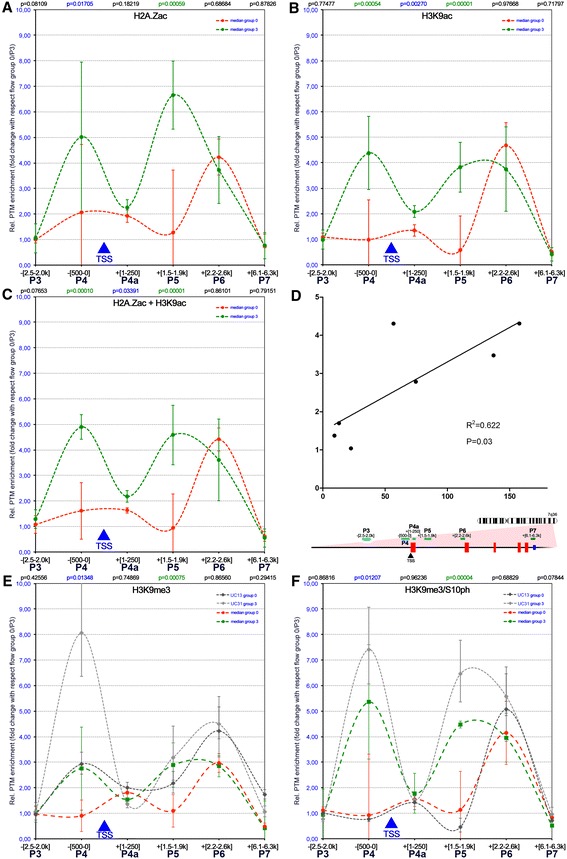
**Dynamic histone acetylation levels adjacent to the transcription start site of NOS3 correlate with impaired placental perfusion and differential NOS3 expression. (A-B)** Histone acetylation patterns differed significantly between patients with high- (green line) and low- (red line) NOS3 mRNA levels. Interestingly, levels of H3K9ac **(A)** as well as H2A.Zac **(B)** were significantly higher surrounding the transcription start site (TSS). **(C-D)** The combined illustration of H3K9ac and H2A.Zac patterns—calculated as mean of both fold changes—demonstrated an even more pronounced effect of histone acetylation at the TSS on NOS3 mRNA levels **(C)** suggesting that both acetylation hallmarks have similar biological consequences. Moreover, the overall level of acetylation around the TSS directly correlated with NOS3 mRNA levels **(D)**. Features of the gene structure cartoon are described in Figure 2. **(E-F)** H3K9me3 levels adjacent to the TSS were higher in patients with high-NOS3 mRNA levels when compared to patients with low-copy number **(E)**—except for one patient with low-NOS3 mRNA copy number who also had a high level of H3K9me3 (UC13). At the same time, patients with high-NOS3 mRNA copy number also showed an increase in H3K9me3/S10ph **(F)**.

We found increased H2A.Zac and H3K9ac levels (amplicons 4 and 5) framing a local depression at the NOS3 TSS (amplicon 4a) in patients with impaired placental perfusion (flow group 3, Figure 3A–C). Adjacent to the TSS and at more distantly located regions up- and downstream, no significant alterations between flow group 0 and flow group 3 could be observed. Remarkably, histone acetylation at two sites flanking the TSS was directly correlated with NOS3 mRNA copy number (*r*^2^ = 0.622, *p* = 0.03; Figure 3D). In two individuals (UC41/UC42) with high-NOS3 mRNA levels, low-H2A.Zac levels at amplicon P4 were associated with high H3K9ac and vice versa at the same site in patient UC37 (Additional file 2: Data 2). Concerning the H3K9me3 distribution, we found higher levels in flow group 3 patients when compared to flow group 0 (Figure 3E). Importantly, the same patients also exhibited significantly higher H3K9me3/S10ph levels, thus possibly counteracting heterochromatin formation, which could provide an explanation for elevated NOS3 mRNA levels in the presence of H3K9me3 in this case (Figure 3F). Remarkably, on an individual base in flow group 3, the exceptionally high-H3K9me3 levels correlated with similar H3K9me3/S10ph increments (UC31, see Figure 3E and Additional file 2: Data 2). Moreover, we made a complementary observation in one flow group 0 patient (UC13) who showed H3K9me3 enrichment at amplicon 4 in the presence of low-H3K9me3/S10ph levels at the same sites (UC13, Figure 3E,F) suggesting that in this case H3K9me3 could be involved in the establishment of a repressive chromatin structure. Taken together, these observations strongly suggest that chromatin plasticity is associated with dynamic PTM combinations at a given site, which may differ between individuals.

### Histone acetylation modifies NOS3 expression in HUAEC in response to trichostatin A treatment entailed by deferred self-attenuation

**Figure 4:**
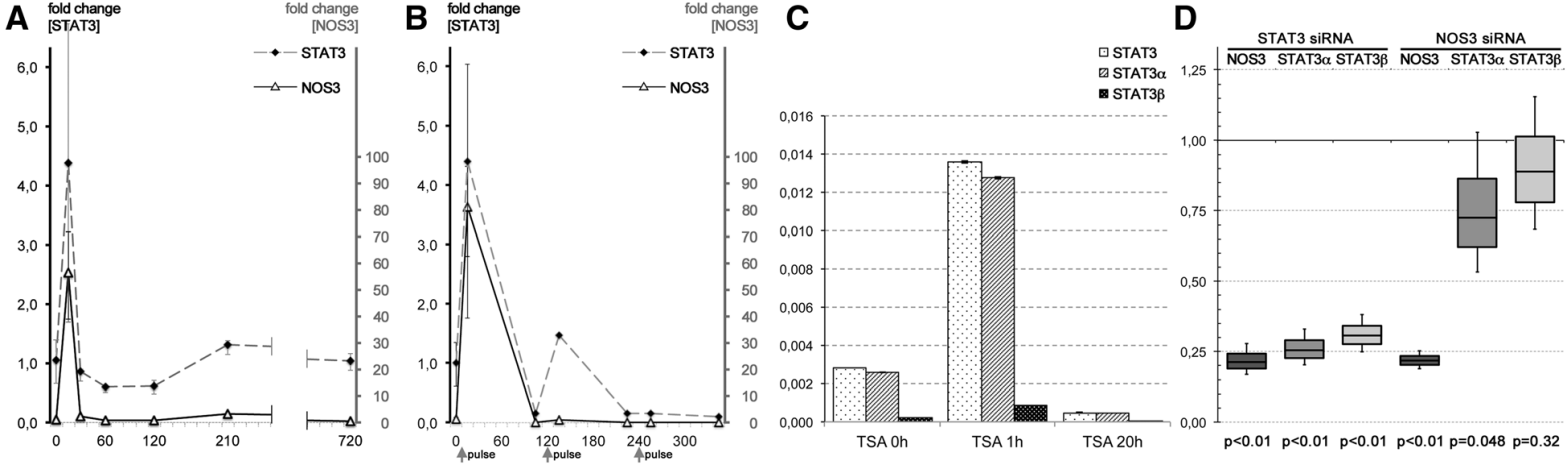
**Histone acetylation changes underlie NOS3 expression in HUAEC in response to trichostatin A treatment and is followed by deferred self-attenuation. (A)** Upon HDAC inhibition via trichostatin A (TSA) in HUAEC, there is a rapid, up to 55-fold increase prior to a steep and long-lasting decrease of NOS3 mRNA levels **(A)**. A similar pattern is seen for STAT3 expression **(A)**. **(B)** Treatment of HUAEC by a restricted TSA pulse leads to an initial boost of NOS3 expression followed by deferred, continuously decreasing amplitudes upon further TSA pulses. **(C)** Expression of STAT3 α not STAT3β is induced by TSA treatment. **(D)** Transfection of HUAEC with siRNA targeted to STAT3 or NOS3 mRNA leads to a significant reduction of NOS3 mRNA levels and vice versa. Thereby, the effect of STAT3 inhibition on NOS3 is much stronger than of NOS3 on STAT3.

To test whether histone acetylation and NOS3 expression are functionally connected, we studied the effects of the HDAC inhibitor trichostatin A (TSA) on NOS3 mRNA synthesis in primary HUAEC. Notably, contrary to naïve expectations, a previous study on HUVEC reported a suppressive effect on NOS3 mRNA synthesis in response to 12-h incubation with TSA [29]. Further, Yan et al. reported that a 27 nt-ncRNA encoded in intron 5 of NOS3 suppresses NOS3 expression via inhibition of STAT3 signalling [23]. With respect to these studies, we hypothesized that inhibition of HDAC activity using TSA would lead to rapidly increased histone acetylation at the NOS3 promoter and increased NOS3 mRNA levels. Consequently, a yet unknown intrinsic negative feedback loop would mediate post-transcriptional reduction of NOS3 mRNA over time. To test this hypothesis, we performed time course experiments in HUAEC supplemented with 1-μM TSA in order to monitor responsive NOS3 and STAT3 expression profiles. As predicted, NOS3 and STAT3 transcription responded in a time-dependent manner with significant and rapid upregulation 15 min after TSA treatment followed by substantial long-lasting downregulation (Figure 4A). The median increase of NOS3 expression directly after TSA treatment was 54.8-fold (range 36.8–77.7), whereby it was 4.4-fold (1.7-10.2) for STAT3 mRNA. To test whether the observed self-attenuation causes resistance against repeated 1-μM TSA pulse-treatments, we performed pulse-release experiments and monitored the responsive expression profiles of NOS3 and STAT3 at discrete time points as described above (Figure 4B). Notably, upregulation of STAT3 in response to the second pulse was substantially reduced (33.51% of pulse 1 response), whereas we only observed a marginal mRNA rise for NOS3 (1.39% of pulse 1 response). Upon a third TSA pulse, no significant fluctuation of both NOS3 (0.09% of pulse 1 response) and STAT3 mRNA levels (3.45% of pulse 1 response) was observed (Figure 4B).

However, evidence for a direct connection between STAT3 signalling and NOS3 remains marginal to date. Since non-redundant biological relevance of two isoforms, Stat3α and Stat3β (encoded by two transcript variants), is controversially discussed [30,31], we decided to discriminate these isoforms. Interestingly, primarily the transcript-variant-encoding Stat3α is expressed in HUAEC cells. This variant was shown to be responsive to TSA treatment in our experiments (Figure 4C). To gain deeper insight into a putative interdependence of STAT3 and NOS3, we analysed whether RNA interference targeting either STAT3 or NOS3 in HUAEC might affect the expression of NOS3 or STAT3α/β (Figure 4D). Quantitative PCR analyses confirmed that both siRNAs led to significant reduction of their target mRNAs. Importantly, levels of STAT3α, but not STAT3β mRNA, were also significantly decreased when NOS3 was targeted by siRNA and vice versa (Figure 4C).

### Site-specific STAT3 enrichment at the NOS3 promoter correlates with increased turnover of H2A.Zac and H3K9ac at the NOS3 TSS in response to hypoxia

**Figure 5:**
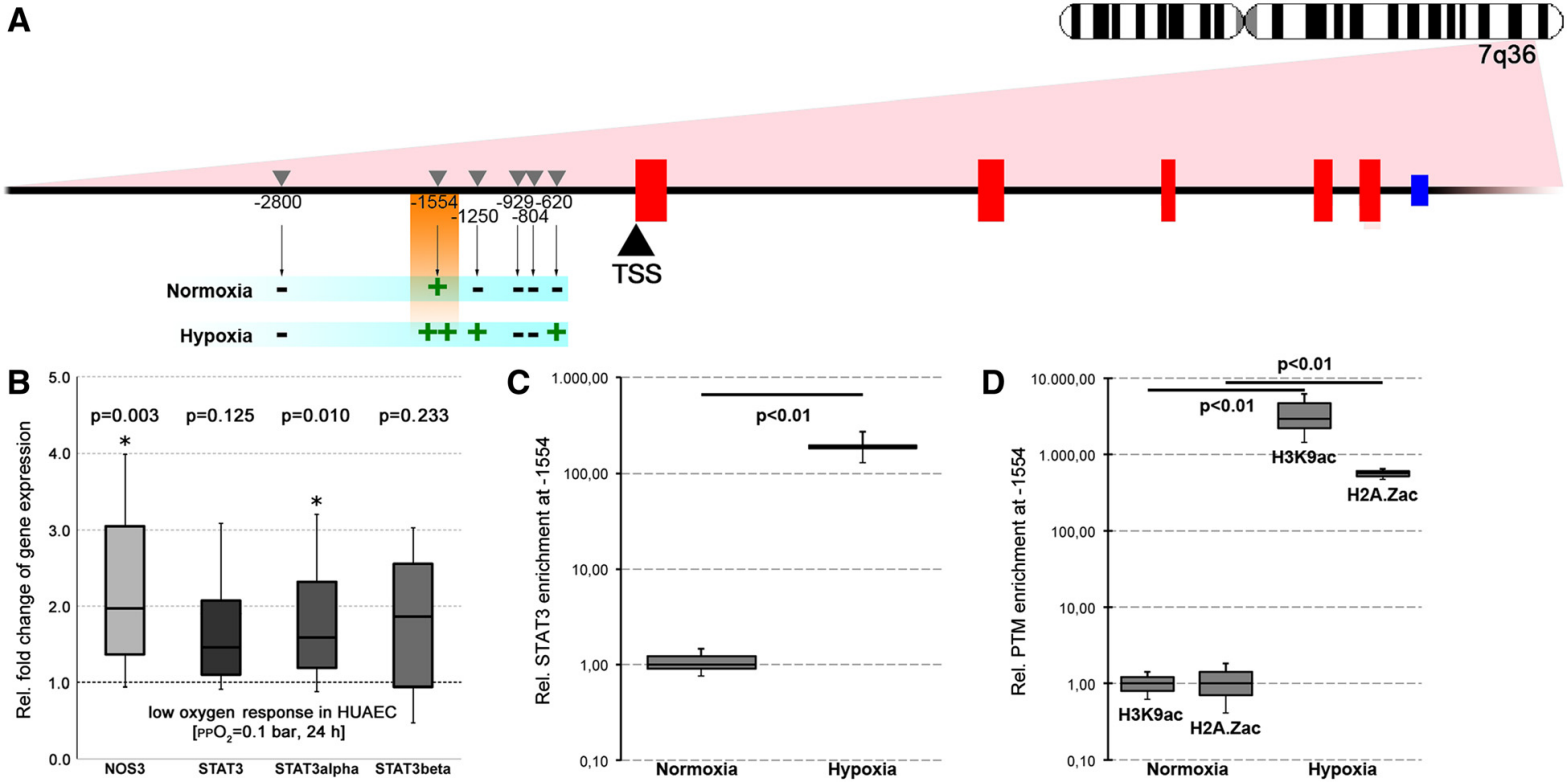
**Hypoxia induces STAT3 and H2A.Zac and H3K9ac turnover at the NOS3 promoter region. (A)** Predicted STAT3 binding site in the NOS3 promoter region based on the STAT3 consensus motifs are shown. During normoxia in human umbilical artery endothelial cells (HUAEC), only one region 1554 bp upstream of the transcription start site (TSS) was enriched in immune precipitates using STAT3 specific antibodies. Enrichment significantly increased during hypoxia with additional sequences being amplified 1,260 and 620 bp upstream of the TSS. **(B)** NOS3 and STAT3, particularly STAT3α expression, is upregulated in HUAEC in response to hypoxia. **(C)** Enrichment of the promoter sequence 1554 upstream of the TSS in STAT3 immune precipitates is upregulated during hypoxia in HUAECs. **(D)** Hypoxia induces a substantial increase in H2A.Zac and H3K9ac turnover at the NOS3 promoter region in HUAECs.

To further analyse the dynamic of NOS3 and STAT3 expression, we made use of an *in vitro* cell culture model simulating placental insufficiency by depriving HUAEC of oxygen for 24 h [16]. This led to a significant increase in mRNA levels for NOS3 and STAT3α, whereas levels of STAT3β remained unchanged (Figure 5B). Importantly, a similar increase in STAT3α mRNA levels was observed in patients with high- versus low-grade placental insufficiency (Additional file 3: Data 3). We then aimed to evaluate the functional relationship between Stat3 protein activity and NOS3 gene expression. Using reported Stat3-binding consensus motifs TT[N]_4_TT, TT[N]_5_TT and TTMNNDAA [32], we identified several potential binding sites in the NOS3 promoter region (Figure 5A, arrows). To quantify possible enrichment of this transcription factor at these sites, we pulled down Stat3 bound to chromatin using specific antibodies followed by qPCR. Under normal culture conditions, only one amplicon containing the predicted −1.554-bp Stat3 binding site upstream of the TSS could be amplified, which thus very likely harbours the primary Stat3 binding site (Figure 5A). Strikingly, during simulated placental insufficiency, Stat3 turnover at the −1.554-bp binding site increased extensively (Figure 5C). Furthermore, two other potential binding sequences, −620 and −1.250 bp, were enriched within the immune complexes (Figure 5A). Last, we used the model to examine the response of histone PTMs associated with the NOS3 TSS to hypoxia. In agreement with our data from clinical samples, the turnover of H2A.Zac and H3K9ac increased significantly during hypoxia (Figure 5D).

### Self-attenuation of NOS3 is a mediated expression of NOS3 intronic 27 nt-ncRNA

**Figure 6:**
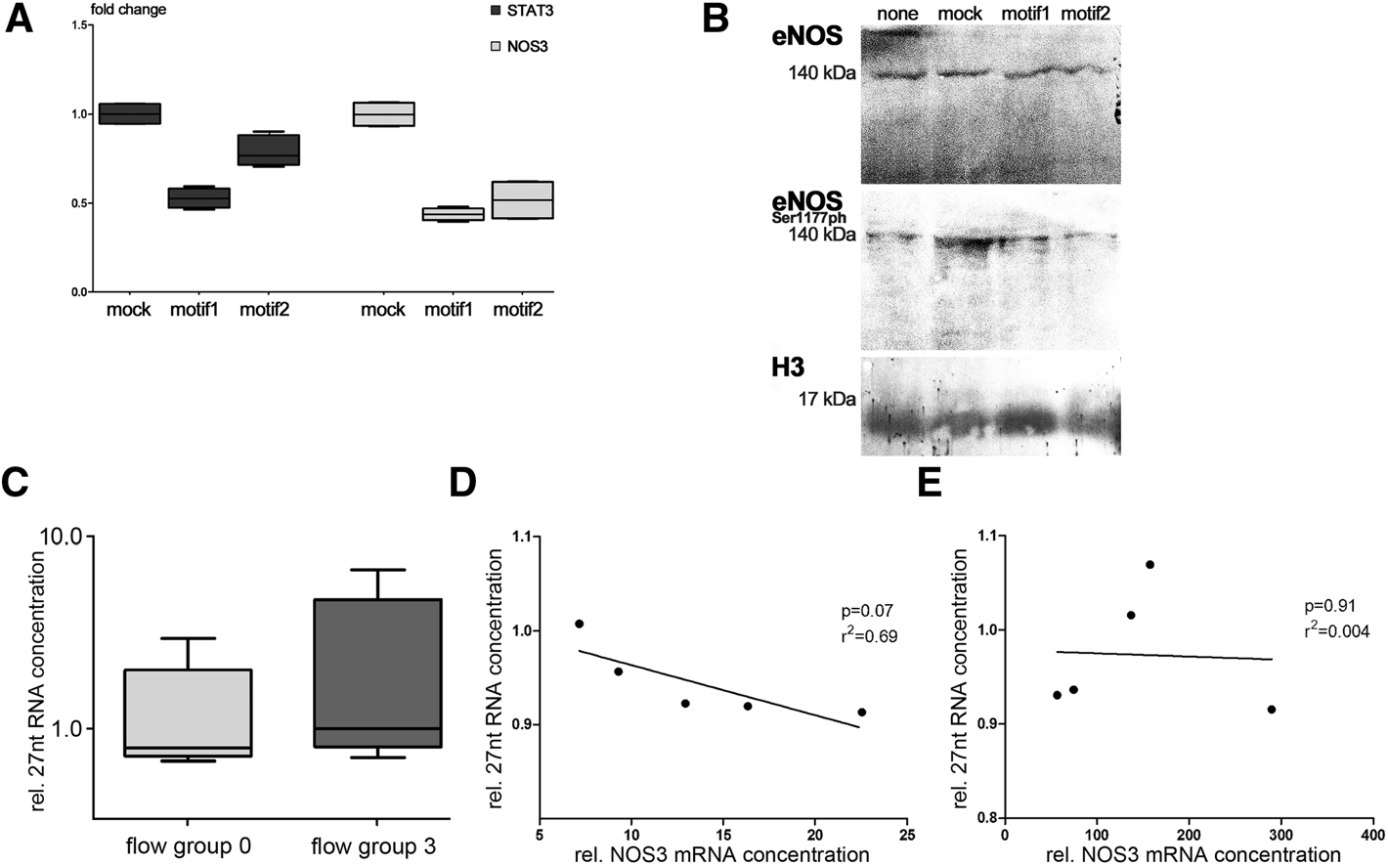
**27 nt-siRNAs target STAT3-3′UTR and correlates with NOS3 mRNA levels in healthy infants.** We used both homologous, slightly different 27 nt-motifs as siRNA for the transfection of HUAEC **(A-B)**. In both motifs, a significant reduction of NOS3 and STAT3 mRNA levels **(A)** and a tendency to lower eNOS and eNOS(Ser1177ph) protein levels **(B)** were observed **(A)** 48 h post-siRNA treatment. No differences in 27 nt-RNA concentrations were found between flow group 0 and 3 **(C)**. Whereas, in infants with normal placental perfusion (flow group 0), 27 nt-RNA enrichment correlated with lower NOS3 mRNA levels **(D)**, no such correlation was found in infants with severe placental insufficiency (flow group 3, **E**).

We next reinvestigated in more detail the influence of the NOS3-intronic 27 nt-ncRNA on both NOS3 and STAT3 expressions. Recently, Zhang and colleagues reported that ectopic expression of tandem decamers or tandem pentamers leads to increased levels of 27 nt-ncRNA in HUAEC, when compared to the expression of tandem tetramers [33]. This suggests that higher tandem-repeat copy numbers lead to increased amounts of mature 27 nt-ncRNA. The population frequency of penta- and tetramers in human alleles exceeds by far hexamers. In our patient’s pool, only one heterozygous hexamer(4c)|tetramer(4a) sample was detected. Basically, the tandem repeats analysed were composed out of variable copy numbers of two homologous 27-nt sequence motifs (motif 1: 5′-GAAGUCUAGACCUGCUGCAGGGG UGAG-3′; motif 2: 5′-GAAGUCUAGACCUGCUGCGGGGGUGAG-3′) leading to a natural total range of 27 nt-ncRNA copy numbers of 8 (4a|4a), 9 (4b|4a) or 10 (4b|4b or 4c/4a) in the studied cohort. To investigate whether both motifs possess redundant potency to suppress NOS3 and/or STAT3 expression, we transfected these oligonucleotides as siRNAs into HUAEC followed by analyses of NOS3 and STAT3 expressions and the availability of eNOS protein (Figure 6A,B). Our data confirm that delivery of both motif 1 and motif 2 leads to diminished availability of NOS3 and STAT3 mRNA (Figure 6A). We also observed a trend towards decreased amounts of eNOS and eNOS(Ser1177ph) in particular the 48-h post-siRNA treatment which however was not statistically significant using semi-quantitative Western blot analysis.

To gain further insights into the clinical relevance of this negative feedback mechanism, we measured 27 nt-ncRNA concentrations in biosamples from our patients. Surprisingly, at first sight, we did not find any differences between 27 nt-ncRNA concentrations when comparing patients with normal (flow group 0) and severely impaired placental perfusion (flow group 3) (Figure 6C). However, in patients without impairment of placental perfusion, enrichment of 27 nt-ncRNA correlated with lower NOS3 mRNA levels and vice versa (*r*^2^ = 0.69, *p* = 0.07; Figure 6D). Importantly, such a correlation could not been found for patients with severe placental insufficiency (*r*^2^ = 0.004, *p* = 0.91; Figure 6E).

### NOS3-intronic 27 nt-ncRNA targets the STAT3-3′-UTR and acts reminiscently of 5′-dominant microRNAs

**Figure 7:**
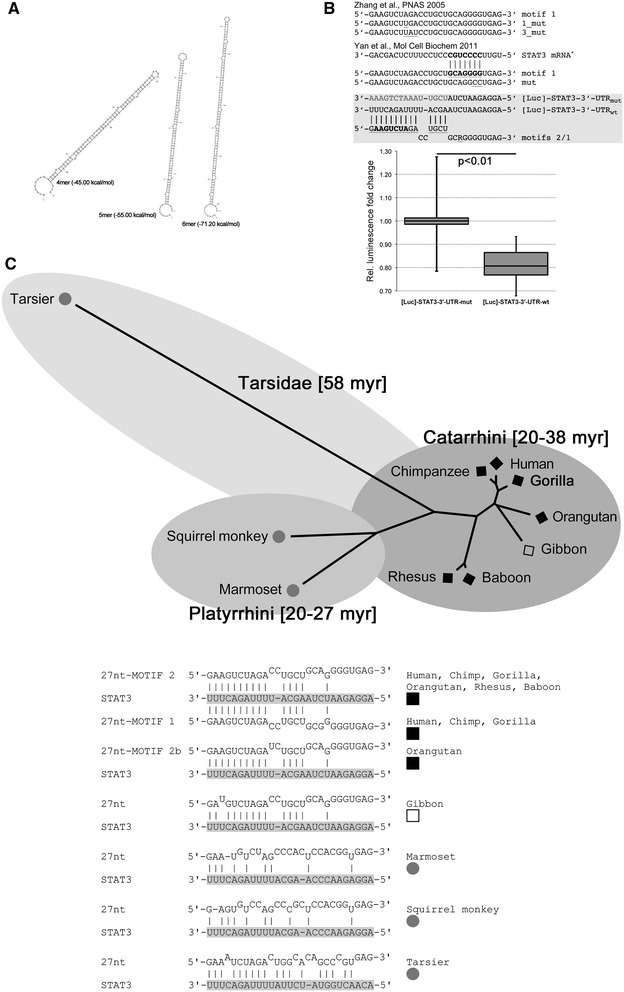
**Targeting of STAT3-3′UTR by 27 nt-siRNAs is reminiscent of miR targeting. (A)** The biogenesis of 27 nt-ncRNA is still enigmatic. This figure shows secondary structure simulations of the different tandem repeats exhibiting regular extended stem-loops. **(B)** The expression of luciferase containing the STAT3 wild-type (wt) 3′-UTR target motif is significantly reduced when compared with luciferase containing a mutated putative STAT3 3′-UTR recognition motif (mut). **(C)** Phylogenetic analyses show that the intronic 27 nt-ncRNA motif is conserved in numerous primate species within the NOS3 gene. Analyses of the corresponding putative STAT3 recognition motifs in those primates suggest a putatively conserved miR recognition mechanism in Catarrhini, whereas less support is given for Platyrrhini and Tarsidae. No evidence for the 27 nt-ncRNA motif was found in species other than primates.

The biogenesis of mature 27 nt-ncRNA remains enigmatic. RNA interference experiments targeting Drosha or Dicer lead to impaired 27 nt-ncRNA maturation, indicating that it depends on the canonical microRNA biogenesis pathway [33]. A precursor has not yet been identified to date. Possibly, the whole intron could act as a long non-coding RNA, from which 27 nt-ncRNAs are further processed. Alternatively, the precursor RNA/pri-miRNA simply consists of tandem-repeated 27 nt-RNA motifs post-transcriptionally processed by an unknown mechanism. We made an attempt to perform secondary structure simulations of the different tandem repeats (Figure 7A) using the mfold tool [34]. In summary, all multimeric 27 nt-RNA tandems exhibited regular stem-loop secondary structures with paired and mismatch positions, regardless of being tetrameric, pentameric or hexameric. Thus, it cannot be excluded that such structures could serve as substrate for Drosha and/or Dicer. However, Drosha-independent non-canonical microRNA biogenesis has also been reported [35]. Two earlier studies proposed that the mature 27 nt-ncRNA acts like a microRNA (miRNA) targeting the 3′-UTR of STAT3 [23,36]. Yan and colleagues argued that a heptameric target sequence is harboured within the 3′-domain of the 27 nt-ncRNA (compare Figure 7B). They reported that mutations within this motif led to a loss-of-function phenotype in a reporter assay. In stark contrast to these results, target recognition of miRNAs usually involves the 5′-domain, which contains the miRNA seed region. This finds convincing support in an elegant study [37] using a *Drosophila* reporter system. The study showed that two major groups exist—5′-dominant as well as 3′-compensatory miRNAs, wherein less complementary 5′-seed base pairing requires stronger 3′ compensatory pairing. Furthermore, the model proposed by Yan and colleagues is opposed by structural restrictions, since Argonaute divides bound miRNAs into functional domains [38]. Mismatches within the miRNA’s seed directly impair binding of the catalytic miRNA-Argonaute complex to its target sequence much more than mismatches in the 3′-supplementary region. We investigated the putative targeting mechanism of the 27 nt-ncRNA. By homology searches, we found an alternative potential target motif in the 3′-UTR of STAT3 perfectly matching as GU wobble to the anchor (1), to the 5′ seed (2–10) and less complementary with the central or 3′-supplementary region (13–16) of the 27 nt-ncRNA. Notably, the variable position between motif 1 and motif 2 (R19) does not interfere with the predicted base pairing (Figure 7B). To test whether the motif found in the 3′-UTR of STAT3 is a target for the putative 27 nt-ncRNA 5′-seed, we performed *in vitro* reporter assays. Therefore, we transfected HELA cells with the pmirGLO Dual-Luciferase miRNA target expression vector containing a large segment of either the wild-type STAT3 3′-UTR or a homologous sequence with mutated putative target site (Figure 7B). Cells were then transfected with the 27 nt-ncRNA oligonucleotides as siRNA, and luciferase expression was analysed by qPCR. The 27 nt-ncRNA caused significantly reduced levels of luciferase mRNAs using the wild-type motif within the 3′-UTR, but not with a mutated motif (Figure 7B). To test for evolutionary conservation of this 27 nt-ncRNA motif, we searched genome databases for primate sequences homologous to NOS3 intron 5. We subsequently performed sequence alignments and identified the 27 nt-motifs. We identified variably sized tandem repeats or singleton motifs within various primate species (Figure 7C, Additional file 4: Data 4), which allows us to date back a common ancestor of the 27 nt-motif at least 58 million years ago [39]. To estimate whether these motifs could encode STAT3 targeting miRNAs, we also extracted the proposed STAT3 target sequence from the databases and analysed potential base pairing mechanisms (Figure 7C). The proposed mechanism with strong 5′-seed and central or 3′-supplementary region matches could also hold for all great ape species, whereas 5′-seed pairing seems to be slightly less complementary in aboriginal primates, such as tarsiers for example. Interestingly, tarsiers exhibit stronger 3′-supplementary pairing to the STAT3 target consistent with a 3′-compensatory miRNA rather than being 5′-dominant. Since most miRNAs are evolutionary conserved, this putative presence of an ancestral 27 nt-ncRNA targeting the STAT3-3′-UTR further supports the relevance of the proposed regulatory mechanism.

## Discussion

In this study, we showed that the foetal response upon alterations in placental perfusion involves differential expression of NOS3 in HUAEC. There was no influence of known genotype variations as described for other disorders in adult patients [40]. Importantly, NOS3 expression correlated with perfusion indices in both the foetal and maternal circulation, thus for the first time linking human clinical data on placental perfusion to molecular changes in the foetus. These *in vivo* data are in line with a recent *in vitro* study demonstrating increased NOS3 activity in HUAEC in response to hypoxia [16].

We also demonstrated that the changes observed in response to placental insufficiency are associated with changes of the epigenetic signatures at the NOS3 gene locus. We noted that the 5meC level at the NOS3 promoter was low in all patients without being correlated with NOS3 expression. This corresponds well with the observation of widespread CpG hypomethylation within the NOS3 promoter in endothelial cells, and hypermethylation in non-endothelial cells [41]. However, we found significant differences in the level of 5hmeC adjacent to the TSS correlating with placental and foetal perfusion indices and NOS3 mRNA levels suggesting a regulatory role for 5hmeC in NOS3 transcription. Mechanistically, the overlap of a local 5meC depression with a 5hmeC peak at the same site might be interpreted as rapid turnover of DNA methylation at this site [7].

Next, we analysed selected histone modifications in relation to NOS3 transcription. Similar to 5hmeC levels, we found increased levels of H3K9ac and H2A.Zac adjacent to the TSS in patients with high-NOS3 mRNA levels who suffered from intrauterine placental insufficiency. Interestingly, in patients with high-NOS3 mRNA levels, H3K9ac and H2A.Zac seemed to be interchangeable with patients having either highly increased turnover of H3K9ac or H2A.Zac or moderately increased acetylation at both sites. This corresponds well to the concept that histone acetylation adjacent to histone-DNA contact sites could directly compensate the positive charge of lysines leading to weaker interactions of the negatively charged DNA phosphate backbone which thus counteracts repressive chromatin configurations [42]. Remarkably, significant changes of histone PTM levels were consistently found at two sites flanking the TSS, whereas the TSS itself behaved relatively inert. This pattern could indicate the existence of at least two well-positioned nucleosomes adjacent to a nucleosome-depleted region in direct proximity to the TSS in NOS3. This observation is also in line with data presented by Fish et al. demonstrating reduced levels of H3K9ac and H2A.Zac in HUVEC associated with decreased eNOS expression *in vitro* [21].

For H3K9me3, which binds snuggly to heterochromatin protein 1 (HP1) and hence promotes heterochromatin formation and gene repression, we found generally low levels in most patients independent of NOS3 mRNA levels. This is in line with the status of a transcriptionally competent gene which is also reflected by the low-5meC levels at the NOS3 promoter observed in all patients. Occasionally and somewhat unexpectedly, we also observed H3K9me3 enrichment in association with high-NOS3 mRNA levels. However, further investigation revealed that concomitantly serine-10 phosphorylation occurred at the same site (H3K9me3/S10ph) in the affected individuals most likely representing a binary methylation/phosphorylation switch, which was reported to counteract HP1 binding and heterochromatin formation, thus disabling the repressive competence of H3K9me3 [28,43]. This status is also somewhat reminiscent of a poised gene associated with bivalent PTM, i.e. the existence of opposing histone modifications at the same nucleosome. Notably, in one patient’s sample exhibiting low-NOS3 mRNA amounts in combination with enriched H3K9me3, H3K9me3/S10ph was absent. Here, H3K9me3 is likely to be involved in the establishment of a repressive chromatin structure at the NOS3 promoter. Overall, the variances in histone acetylation and methylation patterns observed in this study clearly confirm that chromatin signatures involved in the regulation of a specific gene (such as NOS3) act in combination and are interdependent. In clinical specimens from individual patients, they thus need to be interpreted with caution always considering the greater context [44].

In the second part of this study, we aimed to provide deeper mechanistic insights into NOS3 gene regulation using an *in vitro* cell culture model. This was important, because a previous study by Rossig and co-workers reported a marked reduction in NOS3 expression upon treatment of endothelial cells with the HDAC inhibitor TSA for at least 12 h suggesting—in contrast to our *in vivo* findings—a repressive effect of histone acetylation on NOS3 expression [29]. One possible explanation for this contradictory observation could be a negative feedback mechanism counteracting the increased NOS3 transcription enforced by histone acetylation possibly involving a 27 nt-ncRNA encoded in NOS3 intron 5 as proposed earlier [36]. To evaluate this hypothesis, we performed TSA time course experiments in HUAEC also including STAT3 in these analyses, which has been previously linked to NOS3 regulation [23,45]. We could confirm that prolonged TSA treatment indeed resulted in a marked reduction in NOS3 mRNA copy number. However, short-termed treatment significantly increased the NOS3 mRNA copy number. Importantly, a parallel pattern was observed for STAT3 mRNA. Pulse-release experiments with TSA moreover showed that the deferred self-attenuation effect was sustainable. This observation is in agreement with a negative feedback loop entangled with NOS3 expression, which is possibly driven by a putatively co-processed 27 nt-ncRNA encoded in NOS3 intron 5 [36] and involves targeting and cleavage of STAT3 mRNA [23]. To explore this possible connection, we aimed to characterize the functional relationship between NOS3 and STAT3 expressions as well as Stat3 protein function. With respect to known Stat3-binding motifs [32], we identified several potential binding sites within the NOS3 promoter. Using the HUAEC hypoxia model for placental insufficiency [16], we showed that Stat3 selectively bound to one of the predicted sites (namely −1.554 bp upstream of the TSS). Also Stat3 turnover was significantly increased in the course of hypoxia, strongly suggesting a functional connection between Stat3 and NOS3 regulation. In addition, dynamics of NOS3 expression and histone acetylation patterns at the NOS3 TSS in response to hypoxia perfectly corresponded to data obtained from clinical samples.

Next, we explored the mechanism of self-attenuation by intronic 27 nt-ncRNA using its corresponding sequence for RNA interference in HUAEC cultures. As hypothesized, STAT3 and NOS3 mRNA levels decreased significantly in response to treatment with both 27 nt-ncRNA motifs found in the human genome, thus giving further support to the proposed concept of a negative feedback mechanism. Importantly, we were also able to demonstrate that 27 nt-ncRNA levels correlated with NOS3 mRNA levels in patients with normal placental flow whereas this relationship seemed to be dissolved in patients with severe placental insufficiency. This might be an indicator for a more permanent disruption of epigenetic regulatory mechanisms in these infants. If so, this possibly predisposes these patients for vascular disorders later in life.

Since previous hypotheses on the 27 nt-ncRNA targeting mechanism did not seem to hold with regard to current models of miRNA targeting principles, we decided to reinvestigate the underlying mechanisms using an *in vitro* reporter assay as well as phylogenetic analyses. These experiments enabled us to propose a novel hypothesis of STAT3-3′-UTR targeting by the 27 nt-ncRNA, which is reminiscent of 5′-dominant miRNA targeting, including perfect anchor and seed matching, and therefore most probably fulfilling the requirements of target matching and for the assembly of a functional Argonaute-miRNA complex. Furthermore, the proposed mechanism seems to be conserved in a large number of primate species.

## Conclusions

Overall, our study provides comprehensive evidence how an adverse intrauterine *milieu* directly influences human foetal gene expression by means of various levels of gene regulation in vascular endothelial cells, including CpG signalling, chromatin plasticity and non-coding regulatory RNA. Whether the observed changes are reversible or sustained remains an open problem to address. Due to ethical concerns, this question is currently impossible to answer in human individuals given the methodology available. However, epidemiological studies reporting correlation between epigenetic signatures at the NOS3 gene locus in HUAEC and obesity as well as bone mineral content suggest that a hypoxia-induced epigenetic memory state might persist [46]. Such persistence would provide a pathophysiological explanation for the linkage between impaired foetal growth and later vascular abnormalities observed in numerous epidemiological studies [47]. In theory, the epigenetic signature at the NOS3 locus at birth might define an individual baseline level of NOS3 transcription for further life. Alterations of this baseline might subsequently modify the bandwidth of variation in which an individual can respond to adverse events later in life. This in turn could explain the impaired endothelial vascular response observed in patients who were exposed to an adverse intrauterine *milieu*.

## Methods

Written informed consent for human specimen was obtained from all legal guardians. Ethical approval for this study was obtained from the Witten/Herdecke University ethics committee. All work has been conducted according to the principles expressed in the Declaration of Helsinki.

### Nomenclature

The authors of foregoing studies use deviating annotation of the tandemly repeated 27 nt-ncRNA VNTR; namely, it is annotated being encoded within NOS3 intron 4. We refer to human NOS3 mRNA [RefSeq Gene NM_000603] encoded on chromosome 7q36, GenBank ID 4846, which spans a 23.54-kb region from base 150688144 to 150711687 [NC_000007.13]. Thereafter, NOS3 mRNA encoding the canonical transcript variant 1 derives from 27 introns, and the 27-bp tandem repeat is located within intron 5 between exons 5 and 6.

### Patients

Patients were recruited over a 6-month period. Eligible infants obtained Doppler examination of placental and foetal circulation 12–24-h predelivery. GA estimation relied on ultrasound classification before week 14 of gestation. We aimed to depict the whole gestational age range and to include ten patients per flow group (see below). Epidemiological parameters, hospital course data and the outcome were collected. Foetuses with gestational diabetes were excluded since resistance and pulsatility indices differ from normal patients without correlation to foetal growth parameters in these patients [48,49].

### Placental and foetal perfusion assessment

Patients were grouped with respect to blood flow characteristics in the uterine artery (UA), the mediocerebral artery (MCA), and the ductus venosus (DV) as follows [50]:

- group 1: abnormal UA pulsatility index (PI) >2 standard deviations (SD) above mean, or absent UA end-diastolic flow, and normal MCA PI (mean ± 2 SD);
- group 2: abnormal UA PI >2 SD above mean, or absent or reverse UA end-diastolic flow, and abnormal MCA PI (mean < 2 SD) and normal DV PI (mean ± 2 SD);
- group 3: absent or reverse UA end-diastolic flow, and abnormal MCA PI (mean < 2 SD), and abnormal DV PI (mean > 2 SD, a-wave present or absent or reverse end-diastolic flow).

### HUAEC isolation and cell culture

Immediately after delivery, umbilical cords were transported on ice from the maternity ward to the laboratory. HUAEC were separated as described previously [51]. RNA was extracted using TRIzol (Life Technologies) and quantified at 260 nm. RNA integrity was assessed by agarose gel electrophoresis. For cDNA synthesis, we used 500 ng RNA per sample using the QuantiTect Reverse Transcription kit (Qiagen). DNA was isolated by phenol:chloroform:isoamylic alcohol extraction. For *in vitro* experiments, HUAEC (Promocell) were cultivated upon manufacturer’s recommendations. For HDAC inhibition, HUAEC were treated with 1-μM TSA. Hypoxia experiments were performed using a hypoxia incubator chamber (STEMCELL Technologies, Grenoble, France) exposing cells to a ppO_2_ of 0.1 bar corresponding to an oxygen fraction of 10% for 24 h. Control experiments were performed under normoxic conditions (ppO_2_ = 0.21 bar).

### Gene expression analyses

Gene expression analyses were performed using qPCRanalyses on a Rotor-Gene 6000 (Qiagen). For PCR reactions, QuantiTect SYBR Green qPCR Master Mix (Qiagen) containing Hot Start Taq DNA polymerase and SYBR Green was used. Primers were used as listed in Table 2. The expression of genes of interest was normalized to at least three out of five housekeeping genes (BACT, GAPDH, PECAM1, RPL19, VIL1). Following TSA experiments, the mitochondrial ATP6 gene was used for normalization, since interference of this drug with the chromatin state of nuclear housekeeping genes was expected. PCR conditions were as follows: 95°C for 15 min, 40× [95°C for 15 s, 60°C for 30 s]. Melting of PCR product was done using a gradient from 55°C to 95°C rising in 0.5°C increments. For relative comparative quantification of gene expression fold changes, we utilized the ΔΔCt method [52] using at least three housekeeping genes for normalization.

### Genotyping

Polymorphism and zygosity of the NOS3 intron 5 27 nt-ncRNA VNTR was determined by PCR or, in some cases, nested PCR (Table 2). PCR fragments were cloned into the pGEM-T easy vector (Promega) prior to Sanger sequencing (GATC Biotech). Homozygosity was evaluated by at least five replicates.

### Antibodies

Primary antibodies used in this study were as follows: 1. Mouse anti-5meC (Diagenode mAb33D3C15200081), 2. Rat anti-5hmeC (Diagenode mAb633HMC-020), 3. Rabbit anti-H2A.Zac (Diagenode pAb-173-050), 4. Rabbit anti-H3K9ac (Active Motif pAb#39137), 5. Rabbit anti-H3K9me3 (Active Motif pAb#39161), Rabbit anti-H3K9me3S10ph (Diagenode pAbCS-128-100), 5. Rabbit anti-eNOS (Cell Signalling Technologies pAb#9572), 6. Rabbit anti-eNOS Ser1177ph (Cell Signalling Technologies mAbC9C3 #9570), 7. Rabbit anti-H3K4me3 (Diagenode pAbCSP-030-050), 8. Rabbit anti-α-Tubulin (Sigma Aldrich mAbDM1A T9026) and 9. Rabbit anti-Stat3 (D3Z2G, Cell Signalling Technologies mAb#12640).

### (Hydroxy-)methylated DNA immunoprecipitation and qPCR analyses

Analyses of selected CpG-rich sites were performed by (h)MeDIP. Fractions (300 μl) of genomic DNA (100 ng/μl) were sheared on ice by ultrasonic treatment using the Diagenode Bioruptor UCD-200 (12 cycles, 30 s ‘ON’, 30 s ‘OFF’). Most resulting fragments had sizes between ~200–400 bp. These fractions were denatured for 10 min at 95°C and then immunoprecipitated overnight at 4°C in a rotating wheel using 1 μg of mouse monoclonal anti-5meC antibody 33D3 or rat anti-5hmeC antibody. For later comparison, ‘input’ samples were collected. Subsequently, immunocomplexes where separated from suspension using DiaMag protein A-coated magnetic beads (Diagenode), leading to enrichment of methylated DNA fragments. Following several washes, DNA was purified from either ‘input’ or immunoprecipitated samples prior to qPCR analyses. Primer pairs for amplicons corresponding to putative sites with dynamic 5meC or 5hmeC signatures were designed after screening RefSeq sequences for the presence of CpG-rich regions 5,000 bp up- and downstream of the transcription start site using EMBOSS CpGplot (Table 2). Control primers representing an amplicon with low- (GAPDH) or high-5meCpG content (TSH2B) were purchased from Diagenode (pp-1044/pp-1041). PCR conditions were as follows: 95°C for 15 min, 40× [95°C for 15 s, 60°C for 30 s]. Melting of PCR product was done as described above. The recovery of 5meCpG or 5hmeC DNA from total ‘input’ DNA following (h)MeDIP experiments was calculated as follows:

$$ \%\left(\mathrm{I}\mathrm{P}/\mathrm{total}\;\mathrm{input}\right)=\mathrm{A}\mathrm{E}\hat{\mkern6mu} \left[\left(\mathrm{C}\mathrm{t}\left(10\%\mathrm{input}\right)\hbox{-} lo{g}_2\mathrm{D}\mathrm{F}\right)\hbox{-} \mathrm{C}\mathrm{t}\left(\mathrm{I}\mathrm{P}\right)\right]\times 100\% $$

Abbreviations: AE (amplification efficiency); Ct (cycle threshold values obtained from exponential phase of the PCR reaction); the dilution factor (DF) 10 corresponds to 10% ‘input’ sample—thus, the resulting compensatory factor in our experiments was 3.32.

### Chromatin purification and ChIP assays

Chromatin was purified from HUAEC isolated from seven individuals belonging to the low- or high-level NOS3 expression groups. Cells were fixed in PBS/1% formaldehyde for 10 min at room temperature, washed with PBS and incubated with glycine stop solution, prior to additional washing with PBS. Cells and nuclei were then homogenized in ice-cold ChIP buffer (50 mM NaCl; 50 mM Tris–HCl, pH 7.5; 0.1 mM PMSF; 5 mM EDTA; 0.1% SDS) using a Qiagen TissueRuptor device. Following centrifugation for 10 min at 13.000 rpm in a microcentrifuge at 4°C, the supernatant containing the soluble chromatin fraction was collected, and the chromatin concentration was measured at 260 nm using a NanoPhotometer (Implen). Portions of 25-μg (0.1 ng/μl) chromatin were sheared by ultrasonic treatment using a Bioruptor UCD-200 (Diagenode) and 25× [30 s ON/30 s OFF] at position ‘high’. Chromatin fragment size was controlled on agarose gels, and one of the chromatin aliquots was saved as input. For ChIP 25 μg sheared chromatin was incubated with the respective antibody in a rotator for 16 h at 4°C in a total volume of 250 μl ChIP incubation buffer. Subsequently 25 μl protein G magnetic beads (Active Motif) were added and incubated for 4 h at 4°C rotating. Protein G magnetic beads were separated using a magnetic rack and washed repeatedly. To elute DNA fragments enriched by immunoprecipitation, immunocomplexes were incubated with elution buffer (1% SDS, 10 mM EDTA, 50 mM Tris–HCl, (pH 8.1)) for 30 min at 65°C on a shaker. Eluted immunocomplexes were treated with proteinase K. Quantitative PCR analyses were performed using a Rotorgene 6000 (Qiagen). The relative amounts of specifically immunoprecipitated DNA were estimated as ‘percent of input’ and quantified using individual standard curves for each amplicon. Primer pairs were used as described in Table 2.

### RNA interference and reporter assay

For HUAEC transfection, oligonucleotides corresponding to two different 27 nt-ncRNA motifs were used. For transfection into HeLa cells, siRNAs targeted to STAT3 or NOS3 were used. HiPerFect transfection reagent (Qiagen) was used. Effects of 27 nt-ncRNA or siRNAs targeting STAT3 or NOS3 on the expression of NOS3 or STAT3 were assessed in HUAEC 72 h post-transfection by qPCR as described above. For other experiments, we cloned a 1.160-bp DraI/NheI-fragment from the STAT3-3′-UTR containing either the wild-type or a mutated miRNA targeting site into the pmirGLO Dual-Luciferase miRNA target expression vector (Promega). These constructs were transfected into HeLa using Lipofectamine 2000 (Life Technologies) 48 h prior to transfection of siRNAs targeting the [luciferase]-STAT3-3′-UTR. Here, the levels of luciferase mRNAs were measured by qPCR 24 h post-transfection.

### MiR library generation and qPCR.

From selected specimens, we prepared microRNA libraries. Therefore, each sample was tagged with multiplex sequencing barcodes. Total RNA was separated by polyacrylamide gel electrophoresis. Gel fragments corresponding to 15 to 35 nt RNA molecules were cut and RNA was eluted. The obtained small RNA fraction was directly used for the construction of libraries in four steps. Step 1: Ligation of DNA oligonucleotides to the 3′-end of the RNA; Step 2: Ligation of RNA or, respectively, chimeric RNA/DNA oligonucleotides to the 5′-end of RNAs; Step 3: cDNA library synthesis by reverse transcriptase; Step 4: qPCR analyses of the cDNA libraries using pairs of Illumina (San Diego, California, US) index primers, which corresponded to the adapter oligonucleotides used, and a 27 nt-ncRNA-specific primer (5′-tagacctgctgcrggggtgag-3′) were performed as described above.

### Statistical analysis

Mean expression levels were calculated at least from triplicate real-time PCR measurements. In all figures, data are presented as median ± interquartile range (IQR), minimum and maximum, and values for *p* < 0.05 were considered statistically significant unless depicted otherwise. Significance testing was performed using a one-way ANOVA test for values with Gaussian distribution and Mann–Whitney *U* test for values without Gaussian distribution. The Kolmogorov-Smirnov test was utilized to rule out non-Gaussian distribution. All analyses were performed using GraphPad version 5.01 (La Jolla, CA USA).

## Additional files

Additional file 1:
**Data 1.** Interindividual 5meC and 5hmeC signatures at the NOS3 gene locus. Each column colour represents one individual patient analysed. Additional file 2:
**Data 2.** Interindividual histone modification signatures at the NOS3 gene locus. Results of ChIP-qPCR using the same sites as in Figure 3. Each line represents an individual patient. Green lines belong to flow group 3 and red to flow group 0. Additional file 3:
**Data 3.** Relative STAT3α mRNA levels in patients with high- and low-grade placental insufficiency. Results of qPCR. Ten patients were included in flow group 0 and seven in flow group 3. Fold changes were calculated using the Delta Delta CT method. Additional file 4:
**Data 4.** Alignment of primate genomic sequences homologous to human NOS3 intron 5. The 27 nt-motif containing loci are marked by */+ above the aligned sequences.

## Abbreviations

5hmeC: 5-hydroxymethylcytosine
5meC: 5-methylcytosine
ChIP: Chromatin immunoprecipitation
CpG: CG dinucleotide
DNA: Desoxyribonucleic acid
DV: Ductus venosus
eNOS: Endothelial NO-synthase
GA: Gestational age
GAPDH: Glyceraldehyde-3-phosphate dehydrogenase
H2A: Histone 2A
H3: Histone 3
HDAC: Histone deacetylase
HP1: Heterochromatin protein 1
HUAEC: Human umbilical artery endothelial cell
HUVEC: Human umbilical vein endothelial cell
iNOS: Inducible nitric oxide synthase
MCA: Mediocerebral artery
MeDIP: Methylated DNA immunoprecipitation
miRNA: microRNA
ncRNA: Non-coding RNA
NO: Nitric oxide
qPCR: Quantitative real-time polymerase chain reaction
RNA: Ribonucleic acid
TSA: Trichostatin A
TSH2B: Testis-specific histone 2B
TSS: Transcription start site
UA: Uterine artery
VNTR: Variable number tandem repeat
