# Supplementary figures and images for: CpG signalling, H2A.Z/H3 acetylation and microRNA-mediated deferred self-attenuation orchestrate foetal NOS3 expression
Source: Clin Epigenetics. 2015 Feb 8;7(1):9. doi: 10.1186/s13148-014-0042-4 (PMC4333899; doi:10.1186/s13148-014-0042-4)

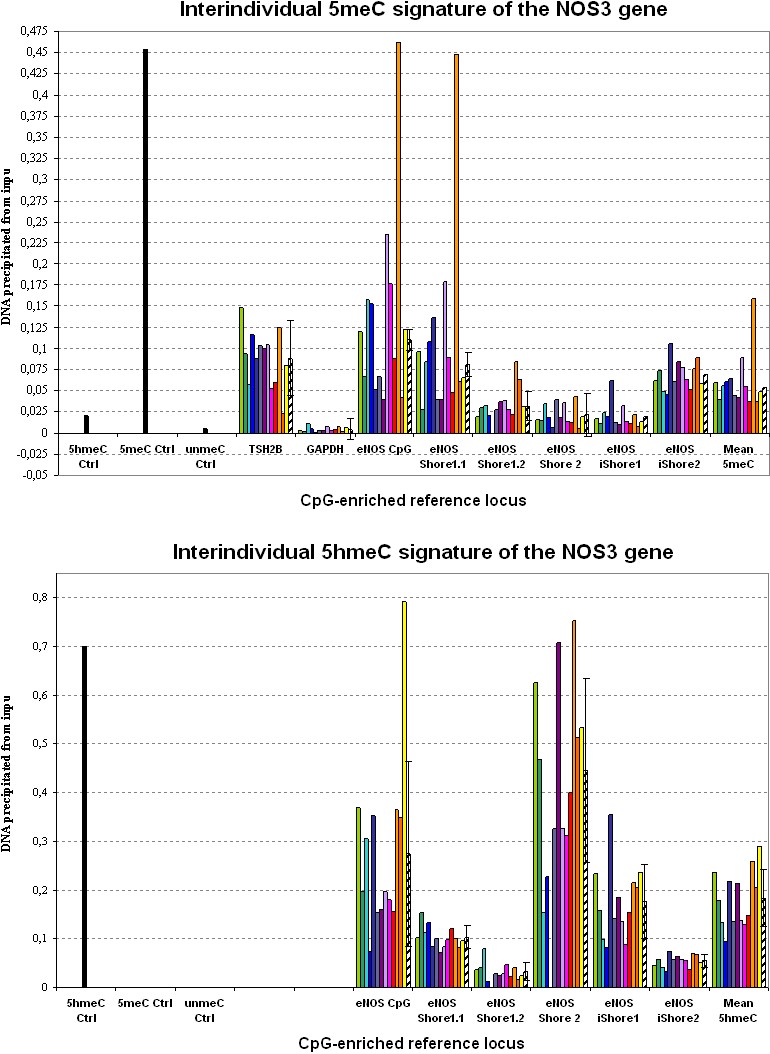

Supplement: Additional file 1: — Data 1. Interindividual 5meC and 5hmeC signatures at the NOS3 gene locus. Each column colour represents one individual patient analysed. [file 13148_2014_42_MOESM1_ESM.tiff]

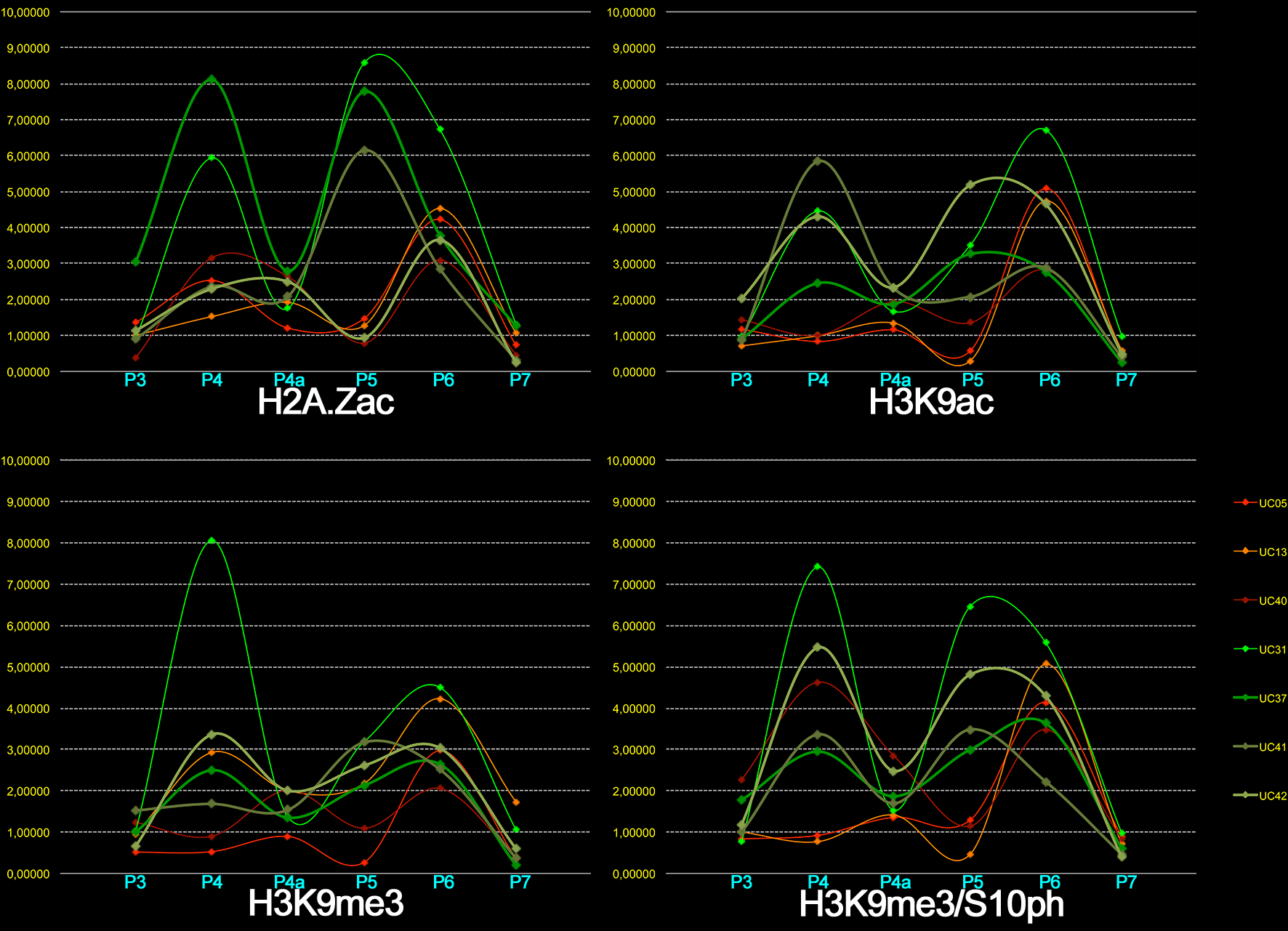

Supplement: Additional file 2: — Data 2. Interindividual histone modification signatures at the NOS3 gene locus. Results of ChIP-qPCR using the same sites as in Figure 3. Each line represents an individual patient. Green lines belong to flow group 3 and red to flow group 0. [file 13148_2014_42_MOESM2_ESM.tiff]

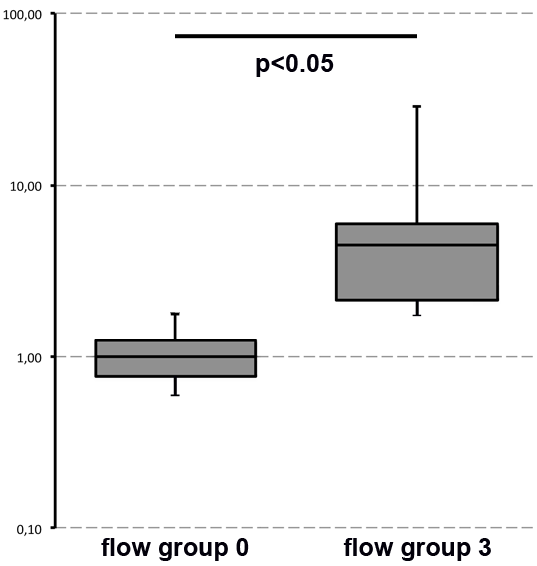

Supplement: Additional file 3: — Data 3. Relative STAT3α mRNA levels in patients with high- and low-grade placental insufficiency. Results of qPCR. Ten patients were included in flow group 0 and seven in flow group 3. Fold changes were calculated using the Delta Delta CT method. [file 13148_2014_42_MOESM3_ESM.tiff]
