# Supplementary material for: CpG signalling, H2A.Z/H3 acetylation and microRNA-mediated deferred self-attenuation orchestrate foetal NOS3 expression
Source: Clin Epigenetics. 2015 Feb 8;7(1):9. doi: 10.1186/s13148-014-0042-4 (PMC4333899; doi:10.1186/s13148-014-0042-4)
Supplement: Additional file 4: — Data 4. Alignment of primate genomic sequences homologous to human NOS3 intron 5. The 27 nt-motif containing loci are marked by */+ above the aligned sequences. [file 13148_2014_42_MOESM4_ESM.pdf]

```
Orangutan/1-1461     1 GTGCGG------------CCGGCCAGCGAGTGAGAGACCCGGGCGCTACCAAAAGGGGAG
Gorilla/1-1349       1 GTGCGG------------CTGGCCAGCGACTGAGAGACCCGGGCGCTACCAAAAGGGGAG
Chimpanzee/1-1404    1 GTGCGG------------CTGGCCAGCGACTGAGAGACCCGGGCGCTACCAAAAGGGGAG
Human/1-1431         1 GTGCGG------------CTGGCCAGCGACTGAGAGACCCGGGCGCTACCAAAAGGGGAG
Gibbon/1-1400        1 GTGCGG------------CCAGCCAGCGAGTGAGAGACCCGGGCGCTACCAAAAGGGGAG
Baboon/1-1380        1 GTGGGGGAAGCTGCAGGTGAGGCCAGCGAGCAAGAGAGCCAGGCACTACTAAAAGGGGAG
Rhesus/1-1376        1 GTGGGGGAAGCTGCAGGTGAGGCCAGCGAGCGAGAGAGCCAGGCACTACTAAAAGGGGAG
Tarsier/1-1346       1 GTGGGGGAAGTTGCAGGTTTGGCCAGTCAGAGGGAAC----TGCGGGACAAAAAGGGGAG
Marmoset/1-1351      1 GTGGGGGAAGCTGCAGGTGTGGCCAGTGAG-----ACCCCAGGCGGGACAAAAAGGGGAG
Squirr mky/1-1332    1 GTGGGGGAAGCTGCAGGTGCGGACAGCGAG-----ACCCCAGGCAGGGCAGAAAGGGGGG

Orangutan/1-1461    49 CGGGGTGGTGGGGCAGTTCCTAAGGCTTCCCGGGGGCTAGGAGGTCCCAAACAATGGGGG
Gorilla/1-1349      49 CGGGGTGGCGGGGCAGTTCCTAAGGCTTCCCGGGGGCTGGGAGGTCCCAAACAATGGGGG
Chimpanzee/1-1404   49 CGGGGTGGCGGGGCAGTTCCTAAGGCTTCCTGGGGGCTGGGAGGTCCCAAACTATGGGGG
Human/1-1431        49 CGGGGTGGCGGGGCAGTTCCTAAGGCTTCCCGGGGGCTGGGAGGTCCCAAACTGTGGGGG
Gibbon/1-1400       49 CGGGGTGGCGGGGCAGTTTCTAAGGCTTCCCCAGGGCTGGGAGGTCCCAAACAATGGGGG
Baboon/1-1380       61 CGGGGTGGCGGGGCAGTTCCTGAGGCTTCCCAGGGACCGGGAGGTCCCAAACAGTGGGGG
Rhesus/1-1376       61 CGGGGTGGCGGGGCAGTTCCTGAGGCTTCCCAGGGACCGGGAGGTCCCAAACAGTGGGGG
Tarsier/1-1346      57 AGGAGGGGTTGAGCAGCTCCTGAGGTTTTCT-GGG--------CTTCCAAGCAGTGGGGG
Marmoset/1-1351     56 CGGGGTGTCGGGGCGGTTCCTGAGGCTTCCCAGGGAATAGGGGGTCTCAAACAGTGGGGG
Squirr mky/1-1332   56 TGGGGTGGC-AGGTAGTTCCTGAGGCTTCCCAGGGACTAGGGGGTCCCAAACAGTGGGGG

Orangutan/1-1461   109 AGATCCTTGCCTGTTCCCTTAGAGACTGGAAAGGTAG-GGAGACC-GCCCCACTCTCAGC
Gorilla/1-1349     109 AGATCCTTGCCTGTTCCCTTAGAGACTGGAAAGGTAG-GAGGACT-GCCCCACCCTCAGC
Chimpanzee/1-1404  109 AGATCCTTGCCTTTTCCCTTAGAGACTGGAAAGGTAG-GGGGACT-GCCCCACCCTCAGC
Human/1-1431       109 AGATCCTTGCCTTTTCCCTTAGAGACTGGAAAGGTAG-GGGGACT-GCCCCACCCTCAGC
Gibbon/1-1400      109 AGATCCTTGCCTGTTCCCTTAGAGACTGGAAAGGTAG-GGGGACC-GCCCCACTCTCAGC
Baboon/1-1380      121 AGATCCTTGCCTGTTCCCTTAGAGACGGGAAAGGTAG-GGGGACC-GCCCCACCCTCAGC
Rhesus/1-1376      121 AGATCCTTGCCTGTTCCCTTAGAGACGGGAAAGGTAG-GGGGACC-GCCCCACCCTCAGC
Tarsier/1-1346     108 AGATCCCTGTCTGTTCCCTCAGAGACTGGAAAAGTAA-GGGGTCCAGCCCTACCCTCAGC
Marmoset/1-1351    116 AGATCGCTGCCTGTTCCCTTAGAGACTAGAAAGGTAGAGGGGCT--GCCCCACCCTCAGC
Squirr mky/1-1332  115 AGATCTCTGCCTGTTCCCTTAGAGACTAGAAAGGTAGAGGGGCT--GCCCCACCCTCAGC

Orangutan/1-1461   167 -ACCCAGGGGAACCTCAGCCCAGTAGTGAGGAC---------------------------
Gorilla/1-1349     167 -ACCCAGGGGAACCTCAGCCCAGTAGTGAAGAC---------------------------
Chimpanzee/1-1404  167 -ACCCAGGGGAACCTCAGCCCAGTAGTGAAGAC---------------------------
Human/1-1431       167 -ACCCAGGGGAACCTCAGCCCAGTAGTGAAGAC---------------------------
Gibbon/1-1400      167 -ACCCAGGGGAACCTCAGCCCAGTAGTGAAGACCTGCTGCTGCAGGCCCCAGATGAGCCC
Baboon/1-1380      179 -ACCCAGGGGAACCTCAACCCAGTAGTGAAGAC---------------------------
Rhesus/1-1376      179 -ACCCAGGGGAACCTCAACCCAGTAGTGAAGAC---------------------------
Tarsier/1-1346     167 TGTCCTGGGGATCCTCAGCCCAGTAGCTAACAG---------------------------
Marmoset/1-1351    174 -ACCCAGGGGAACCTCAGTCCAGTAGTAAAGAC---------------------------
Squirr mky/1-1332  173 -ACCCAGGGGAACCTTAGTCCAGTAGTAAAGAC---------------------------

Orangutan/1-1461   199 ------------------------------------------------------------
Gorilla/1-1349     199 ------------------------------------------------------------
Chimpanzee/1-1404  199 ------------------------------------------------------------
Human/1-1431       199 ------------------------------------------------------------
Gibbon/1-1400      226 CCAGAACTCTTCCTTCTGCCCCCCGAGCTGGTCCTTGAGGTGCCCCTGGAGCACCCCACG
Baboon/1-1380      211 ------------------------------------------------------------
Rhesus/1-1376      211 ------------------------------------------------------------
Tarsier/1-1346     200 ------------------------------------------------------------
Marmoset/1-1351    206 ------------------------------------------------------------
Squirr mky/1-1332  205 ------------------------------------------------------------

Orangutan/1-1461   199 ------------------------------------------------------------
Gorilla/1-1349     199 ------------------------------------------------------------
Chimpanzee/1-1404  199 ------------------------------------------------------------
Human/1-1431       199 ------------------------------------------------------------
Gibbon/1-1400      286 TGAGCACCAAAGGGATTGACTGGGTAGGATGGAGGGGGCCATCCCTGAGCCTCTCAAGAA
Baboon/1-1380      211 ------------------------------------------------------------
```

```
Rhesus/1-1376      211 ------------------------------------------------------------
Tarsier/1-1346     200 ------------------------------------------------------------
Marmoset/1-1351    206 ------------------------------------------------------------
Squirr mky/1-1332  205 ------------------------------------------------------------


Orangutan/1-1461   199 ------------------------------------------------------------
Gorilla/1-1349     199 ------------------------------------------------------------
Chimpanzee/1-1404  199 ------------------------------------------------------------
Human/1-1431       199 ------------------------------------------------------------
Gibbon/1-1400      346 GGGCCTGCAAGGGGGTGCTGATCCCACACCCCAACACCCCCAGGCTGGAGTGGTTTGCAG
Baboon/1-1380      211 ------------------------------------------------------------
Rhesus/1-1376      211 ------------------------------------------------------------
Tarsier/1-1346     200 ------------------------------------------------------------
Marmoset/1-1351    206 ------------------------------------------------------------
Squirr mky/1-1332  205 ------------------------------------------------------------


Orangutan/1-1461   199 -----------------------------------------------CTGGTTATCAGGC
Gorilla/1-1349     199 -----------------------------------------------CTGGTTATCAGGC
Chimpanzee/1-1404  199 -----------------------------------------------CTGGTTATCAGGC
Human/1-1431       199 -----------------------------------------------CTGGTTATCAGGC
Gibbon/1-1400      406 CCCTGGGCCTGCGCTGGTGCACCCTCCCGGCAGTGTCCAACATGCTGCTGGGTATTAGGC
Baboon/1-1380      211 -----------------------------------------------CTGGTTATTAGGC
Rhesus/1-1376      211 -----------------------------------------------CTGGTTATTAGGC
Tarsier/1-1346     200 -----------------------------------------------CTGGTTATTAAGC
Marmoset/1-1351    206 -----------------------------------------------CTGGTTATTAAGC
Squirr mky/1-1332  205 -----------------------------------------------CTGGTTATTAAGC

                                                        ***************************
Orangutan/1-1461   212 CCTATGGTAGTGCCTTGGCTGGAGGAGGGGAAAGAAGTCTAGATCTGCTGCAGGGGTGAG
Gorilla/1-1349     212 CCTATGGTAGTGCCTTGGCTGGAGGAGGGGAAAGAAGTCTAGACCTGCTGCAGGGGTGAG
Chimpanzee/1-1404  212 CCTATGGTAGTGCCTTGGCTGGAGGAGGGGAAAGAAGTCTAGACCTGCTGCAGGGGTGAG
Human/1-1431       212 CCTATGGTAGTGCCTTGGCTGGAGGAGGGGAAAGAAGTCTAGACCTGCTGCAGGGGTGAG
Gibbon/1-1400      466 CCTATGGTAGTGCCTTGGCTAGAGGAGGGGAAA---------------------------
Baboon/1-1380      224 CCTATGGTAGTGCCTTGGCTAGAGGAGGGGAAA---------------------------
Rhesus/1-1376      224 CCTATGGTAGTGCCTTGGCTGGAGGAGGCGAAA---------------------------
Tarsier/1-1346     213 CTTAGGGCAATGCATTGCCCAGAATAGGGGGGGGCG------------------------
Marmoset/1-1351    219 CCTATAGTAGAGCCTTGGCTGGAGGAGGGGA-----------------------------
Squirr mky/1-1332  218 CCTGCGGTAGCGCCTTGGCTGGACGAGGGGA-----------------------------

                       +++++++++++++++++++++++++++***************************++++++
Orangutan/1-1461   272 GAAGTCTAGACCTGCTGCAGGGGTGAGGAAGTCTAGACCTGCTGCAGGGGTGAGGAAGTC
Gorilla/1-1349     272 GAAGTCTAGACCTGCTGCGGGGGTGAG---------------------------------
Chimpanzee/1-1404  272 GAAGTCTAGACCTGCTGCAGGGGTGAGGAAGTCTAGACCTGCTGCAGGGGTGAGGAAGTC
Human/1-1431       272 GAAGTCTAGACCTGCTGCAGGGGTGAGGAAGTCTAGACCTGCTGCAGGGGTGAGGAAGTC
Gibbon/1-1400      499 ------------------------------------------------------------
Baboon/1-1380      257 ------------------------------------------------------------
Rhesus/1-1376      257 ------------------------------------------------------------
Tarsier/1-1346     249 ------------------------------------------------------------
Marmoset/1-1351    250 ------------------------------------------------------------
Squirr mky/1-1332  249 ------------------------------------------------------------

                       +++++++++++++++++++++***************************++++++++++-+
Orangutan/1-1461   332 TAGACCTGCTGCAGGGGTGAGGAAGTCTAGACCTGCTGCAGGGGTGAGGAAGTCTAGA-C
Gorilla/1-1349     299 ------------------------------------------------------------
Chimpanzee/1-1404  332 TAGACCTGCTGCGGGGGTGAG---------------------------------------
Human/1-1431       332 TAGACCTGCTGCGGGGGTGAG---------------------------GAAGTCTAGA-C
Gibbon/1-1400      499 ------------------------------------------------GATGTCTAGA-C
Baboon/1-1380      257 ------------------------------------------------GAAGTCTAGA-C
Rhesus/1-1376      257 ------------------------------------------------GAAGTCTAGA-C
Tarsier/1-1346     249 ------------------------------------------------GAAATCTAGA-C
Marmoset/1-1351    250 ------------------------------------------------GAA-TGTCTAGC
Squirr mky/1-1332  249 ------------------------------------------------GA-GTGTCCAGC
```

```
                         ++-++++++++++++++
Orangutan/1-1461    391 CT-GCTGCAGGGGTGAGGACA---------------CCTGGGCGGTGC----TGTCAGTA
Gorilla/1-1349      299 -----------------GACAGCTGAGCGGAGCTTCCCTGGGCGGTGC----TGTCAGTA
Chimpanzee/1-1404   353 -----------------GACAGCTGAGCGGAGCTTCCCTGGGCGGTGC----TGTCAGTA
Human/1-1431        364 CT-GCTGCGGGGGTGAGGACAGCTGAGCGGAGCTTCCCTGGGCGGTGC----TGTCAGTA
Gibbon/1-1400       510 CT-GCTGCAGGGGTGAGGACAGCTGAGTGGAGCTTCCCTGGGCGGTGC----TGTCAGTA
Baboon/1-1380       268 CT-GCTGCAGGGGTGAGGACAGCTGAGCGGAGCTTCCTTGGGCGGTTC----TGTCAGTA
Rhesus/1-1376       268 CT-GCTGCAGGGGTGAGGACAGCTGAGCGGAGCTTCCTTGGGCGGTTC----TGTCAGTA
Tarsier/1-1346      260 -TGGCACAGCCCGTGAGGATAGCTGAGCAGTGCTTCCCTGGGGATGCTG---TTCAAGTA
Marmoset/1-1351     261 -CCACTCCACGG-TGAGGACAGCTGGGTGCAGCTTCCCCGGGTGGTGGGTGCCGTCAGTA
Squirr mky/1-1332   260 -CCGCTCCACGG-TGAGGACAGCTGGGCGCAGCTTCCCTGGGCGGTGGGTGGTGTCAGTA
Orangutan/1-1461    431 GCAGGAGCAGCCTCCTGGAAAG-CCCTGGCTGCTGCTTCTCCCCCAAGAGAGAAGGCTT
Gorilla/1-1349      338 GCAGGAGCAGCCTCCTGGAAAG-CCCTGGCTGCTGCTTCTCCCCCAAGAGAGAAGGCTT
Chimpanzee/1-1404   392 GCAGGAGCAGCCTCCTGGAAAG-CCCTGGCTGCTGCTTCTCCCCCAAGAGAGAAGGCTT
Human/1-1431        419 GCAGGAGCAGCCTCCTGGAAAG-CCCTGGCTGCTGCTTCTCCCCCAAGAGAGAAGGCTT
Gibbon/1-1400       565 GCAGGAGCAGCCTCCTGGAAAG-CCCTGGCTGCTGCTTCTCCCCCAAGAGAGAAGGCTT
Baboon/1-1380       323 GCAGGAGCAGCCTCCTGGAAA-G-CCCTGGCTGCTGCTTCTCCCCCAAGGGAGAAGGCTT
Rhesus/1-1376       323 GCAGGAGCAGCCTCCTGGAAA-G-CCCTGGCTGCTGCTTCTCCCCCAAGGGAGAAGGCTT
Tarsier/1-1346      316 ACAGGAGCAGCCTCCTGGAAAGGGCCCTGCCTGCTGCTTCCCTG----------AGG---
Marmoset/1-1351     319 GCAGGAGCGGCCTCCTAGAAAG-CCCTGGCTGCTGCTTCTCCCC----------------
Squirr mky/1-1332   318 GCAGGAGCGGCCTCCTGGAAAG-CCCTGGCTGCTGCTTCTCCCC----------------


Orangutan/1-1461    490 CTCCTGCCAGGCCAGTCCAGTGCAGCCCCTCACCGACACCCGCTGCTC----CCCCAGTT
Gorilla/1-1349      397 CTCCCGCCAGGCCAGTCCAGTGCAGCCCCTCACCCACACCCACTGCTA----CCCCAGTT
Chimpanzee/1-1404   451 CTCCCGCCAGGCCAGTCCAGTGCAGCCCCTCACCCACACCCACTGCTA----CCCCAGTT
Human/1-1431        478 CTCCCGCCAGGCCAGTCCAGTGCAGCCCCTCACCCACACCCACTGCTA----CCCCAGTT
Gibbon/1-1400       624 CTCCCGCCAGGCCAGTCCAGTCCAGCCCCTCACCCATACCCACTGCTC----CCCTAGTT
Baboon/1-1380       381 CTCCTGCTAGGCCAGTCCAGTACAGCCCCTCACCCACACCCGCTGCTC----CCCCAGTT
Rhesus/1-1376       381 CTCCTGCTAGGCCAGTCCAGTGCAGCCCCTCACCCACACCCGCTGCTC----CCCCAGTT
Tarsier/1-1346      363 -----GTCAGGTCTGCCCAACCCGTGCCCTCACCCACACCCATTGCTCCCCTCCCCAGTT
Marmoset/1-1351     362 -----ACCAGGCCAGCCCAGTGCAGCCCCTCACCCACACCCGGTGCTCC---CCCCAGCT
Squirr mky/1-1332   361 -----ACCAGGCCAGCCCAGTGCAGCCCCTCACCCACACCCAGTGCTCC---CCACAGGT


Orangutan/1-1461    546 CCCCTGCCTCGGCCCGCACCCGCCCTCACACCCCAGCCCACAGACTCGGGGCTGGCCTTG
Gorilla/1-1349      453 CCCCTGCTTCGGCCCGCACCCTCCCTCACACCCCAGCCCACAGACTCGGGGCTGGCCTTG
Chimpanzee/1-1404   507 CCCCTGCTTCGGCCCGCACCCTCCCTCACACCCCAGCCCACAGACTCGGGGCTGGCCTTG
Human/1-1431        534 CCCCTGCTTCGGCCCGCACCCTCCCTCACACCCCAGCCCACAGACTCGGGGCTGGCCTTA
Gibbon/1-1400       680 CCCCTGCCTCAGCCCGCACCCTCCCTCACACCCCAGCCCACAGACTCGGGGCTGGCCTTG
Baboon/1-1380       437 CCCCTGCCTCGACGTGCACCCTCCCTCACACCCCAGCCCACAGACTCGGGGCTGGCCTTG
Rhesus/1-1376       437 CCCCTGCCTCGACGTGCACCCTCCCTCACACCCCAGCCCACAGACTCGGGGCTAGCCTTG
Tarsier/1-1346      418 CTCCTGCCTCGACCTGGACCC------------------GTAGGCTCAGGGCTGGCCTGG
Marmoset/1-1351     414 CCCCTGCCCCAGCCCGCACCC--CCTCACACCCCAGCCCACAGACTCAGAGCTGGCCTCA
Squirr mky/1-1332   413 CCCCTGCCCCAGCCCACACCC-CCCACACACCTCAGCCCACAGACTCAGTGGTGGCCTCG


Orangutan/1-1461    606 GTTACTGGAAC-GCCTGTGACCACAGCACTAAGAGAAGCAAGCTGCCCCATGGGGAAC-T
Gorilla/1-1349      513 GTTACTGGAAC-GCCTGTGACCACAGCACTAAGAGAAGCAAGCTGCCCCATGGGGGAC-T
Chimpanzee/1-1404   567 GTTACTGGAAC-GCCTGTGACCACAGCACTAAGAGAAGCAAGCTGCCCCATGGGGGAC-T
Human/1-1431        594 GTTACTGGAAC-GCCTGTGACCACAGCACTAAGAGAAGCAAGCTGCCCCATGGGGGAC-T
Gibbon/1-1400       740 GTTACTGGAAT-GCTTGTGACCACAGTACTAAGAGAAGCAAGCTGCCCCATGGGGGAC-T
Baboon/1-1380       497 GTTACTGGAACAGCCTGTGACCACAGCACTGAGAGAAGCAAGCTGCCCCATGGGGGAC-T
Rhesus/1-1376       497 GTTACTGGAACAGCCTGTGAGCACAGCACTGAGAGAAGCAAGCTGCCCCATGGGGGAC-T
Tarsier/1-1346      460 GTTATTGAG-CAGCCTGTGCCCATGGCACTAAGAGAAGCAAGCTGACCCATAAGGGAA--
Marmoset/1-1351     472 GTTGCTAGAACAGCCTGTGACCACAGCACTAAGAGAAGCAAGCTGCCCCATGGGGGAA-C
Squirr mky/1-1332   472 ATCACTAGAACAGCCTGTGCCCACGGCACTAAGAGAAGCAAGCTGCCCCATGGGGGAAAC


Orangutan/1-1461    664 -GGTCCCACGGCCTCGGCCTCCTTCACCGTC--ACTGGCCGCCAAA----GAGTTTGAAA
Gorilla/1-1349      571 TGGTCCCATGGCCTTGGCCTCCTTCACCATC--ACTGGCCGCCAAA----GAGTTTGAAA
Chimpanzee/1-1404   625 TGGTCCCATGGCCTTGGCCTCCTTCACCATC--ACTGGCCACCAAA----GAGTTTGAAA
Human/1-1431        652 TGGTCCCATGGCCTTGGCCTCCTTCACCATC--ACTGGCCGCCAAA----GAGTTTGAAA
Gibbon/1-1400       798 TG----------------------------------------------------------
Baboon/1-1380       556 TGGTCCCATGGCCTTGGCCTCCTTCACCATC--ACTGGCCGCCAAA----GAGTTTGAAA
Rhesus/1-1376       556 TGGTCCCATGGCCTTGGCCTCCTTCACCATC--ACTGGCCGCCAAA----GAGTTTGAAA
```

```
Tarsier/1-1346      517 TCAGCCCATGATCTTGGCCTCCTTGGCTGTA--ACTGGCCACCAAA----GAACTTGGAG
Marmoset/1-1351     531 TGCACCTACAGCCTTGACCTCCTTCACTGGC---CTGGCCGCCAAA----GAGTTTGGAA
Squirr mky/1-1332   532 TGCACCTACGGCCTTGACCTTCTCCGCCACTGGACTGGCCACCAAAAAAAGAGTTTGGAA

Orangutan/1-1461    717 TAAAGCCACGTGCCCAGTGAATCCCAGAGGAACCTCAAATAAAATAAAAACAATCCTATC
Gorilla/1-1349      625 TAAAGCCACGTGCCCAGTGAATCCCAAAGGAACCTCAAATAAAATAAAAACAATCCTATC
Chimpanzee/1-1404   679 TAAAGCCACGTGCCCAGTGAATCCCAAAGGAACCTCAAATAAAATAAAAACAATCCTATC
Human/1-1431        706 TAAAGCCACGTGCCCAGTGAATCCCAAAGGAACCTCAACTAAAATAAAAACAATCCTATC
Gibbon/1-1400       800 ------------------------------------------------------------
Baboon/1-1380       610 TAAAGCCACGTGCCCAGTGAATCCCAAAGGAGCCTCAAATAAAATAAAAACAATCCTATC
Rhesus/1-1376       610 TAAAGCCACGTGCCCAGTGAATCCCAAAGGAGCCTCAAATAAAATAAAAACAATCCTATC
Tarsier/1-1346      571 TAAAGCCACATGCTCAGTAAATCCCAAAGAAGTCTCAAATAAAACAAGAATAATCCTGTC
Marmoset/1-1351     584 TAAAGCCTTATGCCCAGTGACTCCCAAAGCAGTCTCCAGTAAAACACAAACTATCCAATC
Squirr mky/1-1332   592 TAAAGCCTCGTGCCTAGTGAATCCCAAAGGAGTCTCGAGTAAAATAAAAACAATCCTATC
Orangutan/1-1461    777 TGACACTTGCCTGACCCTCT-AAGTCGTTCAAAGCTTTAGCTCAACTTTGATCC-ATCTG
Gorilla/1-1349      685 TGACACTTGCCTGACCCTCT-AAGTCGTTCAAAGCTTTAGCTCAACTTCGATCC-ATCTG
Chimpanzee/1-1404   739 TGACACTTGCCTGACCCTCT-AAGTCATTCAAAGCTTTAGCTCAACTTCGATCC-ATCTG
Human/1-1431        766 TGACACTTGCCTGACCCTCT-AAGTCATTCAAAGCTTTAGCTCAACTTCGATCC-ATCTG
Gibbon/1-1400       800 ------------------------------------------------------------
Baboon/1-1380       670 TGACACTTGCCTGACACTCT-AAGTTGTTCAAAGCTTTAACTCAACTTTGATCC-ATCTG
Rhesus/1-1376       670 TGACACTTGCCTGACACTCT-AAGTTGTTCAAAGCTTTAACTCAACTTTGATCC-ATCTG
Tarsier/1-1346      631 TGACACTTGCCTAAC--TCTTAAGTTCTTCAAAGCTTTAGCTCAACTTTGAACCCATCTG
Marmoset/1-1351     644 CGACACCTGCCTGACCCTCT-AAGTCGTTCAAAGCTTTAGCTCAACTTTGATCCC-TTTG
Squirr mky/1-1332   652 TGACACCTGCCTGACCCTCT-AAGTTGTTCAAAGCTTTAGCTCAACTTTGATCCC-TCTG

Orangutan/1-1461    835 AGTTGCCATAGTGGACCCCACTCAGAGCTGCGTCCCTCCCTTGACCCCAGGCTGGT---C
Gorilla/1-1349      743 AGCTGCCATAGTGGACCCCACTCAGAGCTGCGTCCCTCCCTTGACCCCAGGTTGGT---C
Chimpanzee/1-1404   797 AGCTGCCATAGTGGACCCCACTCAGAGCTGCGTCCCTCCCTTGACCCCAGGTTGGT---C
Human/1-1431        824 AGCTGCCATAGTGGACCCCACTCAGAGCTGCGTCCCTCCCTTGACCCCAGGTTGGT---C
Gibbon/1-1400       800 --------------------------CTGCGTCCCTCCCTTGACCCCAGGCTGGTGGTC
Baboon/1-1380       728 AGCTGCCATAGTGGACCCCACTCAGAGCTGCGTCCCTCCCTTGACCCCAGGCTGGT---C
Rhesus/1-1376       728 AGCTGCCATAGTGGACCCCACTCAGAGCTGCGTCCCTCCCTTGACCCCAGGCTGGT---C
Tarsier/1-1346      689 AGCTTCCATAATGAACCCTACTCATAGCTATGTCCCTCCCTTGATCCCAGGCCAGT---C
Marmoset/1-1351     702 AGCTGCCATAGTGGGCCCCACTCAGAGCTGTGT--------------------------C
Squirr mky/1-1332   710 AGCCGCCATAGTGGGCCCCACTCAGAACTTCGT--------------------------C

Orangutan/1-1461    892 CCTGCCACTCCCCTGCCCCTGTCACTGACACACGTTTCCTCCTCCCTCAGGCAGGAGTGG
Gorilla/1-1349      800 CCTGCCACTCCCCTGCCCCTGTCACTGACACACGTTTCCTCCTCCCTCAGGCAGGAGTGG
Chimpanzee/1-1404   854 CCTGCCACTCCCCTGCCCCTGTCACTGACACATGTTTCCTCCTCCCTCAGGCAGGAGTGG
Human/1-1431        881 CCTGCCACTCCCCTGCCCCTGTCACTGACACATGTTTCCTCCTCCCTCAGGCAGGAGTGG
Gibbon/1-1400       833 CCTGCCACTCCCCTGCCCCTGTCACTGACACACGTTTCCTCCTCCGTCAGGCAGGAGTGG
Baboon/1-1380       785 CCTGCCACTCCCCTGCCCCTGTCACTGGCACATGTTTCCCCCTCCCTCAGGCAAGAGTGG
Rhesus/1-1376       785 CCTGCCACTCCCCTGCCCCTGTCACTGGCACATGTTTCCTCCTCC-TCAGGCAAGAGTGG
Tarsier/1-1346      746 CCTGCCA--CCCCTACCTCTGTCACCAACACATGTTTCCTCCTCCCTCGGGCAGGAATGA
Marmoset/1-1351     736 CCTGCCACTCCCCTGCTCCTGTCACTGACATGTGTTTCCTCCTCCCTCAGGCAGGAGTGG
Squirr mky/1-1332   744 CCTGCCACTCCCCTGCCCCTGTCACTGACACGTGTTTCCTCCTCCCTCAGGCAGGAGTGT

Orangutan/1-1461    952 GACCTCCCAGCCTCCTCCTGGGGCCTCCACTCA---------------------------
Gorilla/1-1349      860 -ACCTCCCAGCCTCCTCCTGCGGCCTCCACTCA---------------------------
Chimpanzee/1-1404   914 GACCTCCCAGCCTCCTCCTGGGGCCTCCACTCA---------------------------
Human/1-1431        941 GACCTCCCAGCCTCCTCCTGGGGCCTCCACTCA---------------------------
Gibbon/1-1400       893 GACCTCCCAGCCTCCTCCTGGGGCCTCCACTCA---------------------------
Baboon/1-1380       845 GACCTCCCAGCCTCCTCCTGGGGCCTCCACTCA-GCCAGCCAAGACAGCTCCTCCAAGCC
Rhesus/1-1376       844 GACCTCCCAGCCTCCTCCTGGGGCCTCCACTCA-GCCAGCCAAGACAGCTCCTCCAAGCC
Tarsier/1-1346      804 GACCTCCCAGTCTCCT---GGGGCAT-CACTCAAGTCAGCCAAGGCTT-TCCTGG---CA
Marmoset/1-1351     796 GACCTCCCAGCCTCCTCCTGGGGCCTCCACTCA-GCCAGCCAAGACAGCTCCTCCAAGCA
Squirr mky/1-1332   804 GACCTCCCAGCCTCCTTCTGGGGCCTCCACTCA-GCCAGCTAAGACAGCTCCTCCAAGCG

Orangutan/1-1461    985 ---GAATGTCAGGATGAGCAGGGTCCTAGGAGGCCCCCGGTGCAGCCTTCCC-TTCCCAC
Gorilla/1-1349      892 ---GAATGTCAGGATGAGCAGGGTCCTAGGAGGCCTTTGGTGCAGCCTTCCC-TTCCCAC
Chimpanzee/1-1404   947 ---GAATGTCGGGATGAGCAGGGTCCTAGGAGGCCTCTGGTGCAGCCTTCCC-TTCCCAC
```

```
Human/1-1431        974 ---GAATGTCAGGATGAGCAGGGTCCTAGGAGGCCTCTGGTGCAGCCTTCCC-TTCCCAC
Gibbon/1-1400       926 ---GAATGTCAGGATGAGCAGGGTCCTAGGAGGCCCCTGGTGCAGCCTTCCC-TTCCCAC
Baboon/1-1380       904 CTAGAATGTCAGGATGAGCAGGGTCCTAGGAGGCCCCTGGTGCAGCCTTCCC-TTCCTAC
Rhesus/1-1376       903 CTGGAATGTCAGGATGAGCAGGGTCCTAGGAGGCCCCTGGTGCAGCCTTCCC-TTCCTAC
Tarsier/1-1346      856 TCAGAATGTCAGCGTTGGCAGGGTCCCTGGAGGCCACTTAGTACACCACCCCCTTCCTAT
Marmoset/1-1351     855 CTAGAATGTCAGGATGAGCAGCATCCCAGAAGGCCTCCTGTGCAGGCTTCCC-TTCCCAT
Squirr mky/1-1332   863 CTAGAATGTCAGGATGAGCAGCGTCCCAGGAGGCCCCCTGTGTAACCTTCCC-TTCCCAT


Orangutan/1-1461   1041 CATCCATGTG-CTCAAAGAGAATAAGATGGCTGGCTGGGGTCACCCGTCCTTTCTTGAAT
Gorilla/1-1349      948 CATCCATGTG-CTCAAAGAGAAT------------------CACCCGTCCTTTCTTGAAT
Chimpanzee/1-1404  1003 CATCCATGTG-CTCAAAGAGAAT------------------CACCCGTCCTTTCTTGAAT
Human/1-1431       1030 CATCCATGTG-CTCAAAGAGAAT------------------CACCCGTCCTTTCTTGAAT
Gibbon/1-1400       982 CATCCATGTG-CTCAAAGAGAATCAGATGGCTGGCTGGG-TCACCCGTCCTTTCTTGAAT
Baboon/1-1380       963 CATCCATGTG-CTCAAATAAAATCAGATGGCTGGCTGGGGTCACCCGTCATTTCTTGAGT
Rhesus/1-1376       962 CATCCATGTG-CTCAAATAAAATCAGATGGCTGGCTGGGGTCACCCGTCATTTCTTGAGT
Tarsier/1-1346      916 CATCAATGTG-CTCAAGGAAAGTCAGATGGCCAA-TGGGGTCACCTGTCTTTGCTTGAGC
Marmoset/1-1351     914 CGTTCATGTGATTCTCAGAGAATCAGATGGCTGCCTGGGGTCACCTGTCCCTTCTTGAGT
Squirr mky/1-1332   922 CATCCGTGTGATTCTCAGAGAATCAGATGGCTGCCTGGGGTCACCTGTCTCTTCTTGAGT
Orangutan/1-1461   1100 GCCATGGATCATGGGGGATTTGCTGCCCACACTCCTAGGCGGTCTCTTAGACATCCGTTG
Gorilla/1-1349      989 GCCATGGATCATGGGGGATTTGCTGCCCACACTCCTAGGCGGCCTCTTAGACATCCGTTG
Chimpanzee/1-1404  1044 GCCATGGATCATGGGGGATTTGCTGCCCACACTCCTAGGCGGCCTCTTAGACATCCGTTG
Human/1-1431       1071 GCCATGGATCATGGGGGATTTGCTGCCCACACTCCTAGGCGGCCTCTTAGACATCCGTTG
Gibbon/1-1400      1040 GCCACGGATCATGGGGGATTTGCTGCCCACACTCCTAGGCGGCCTCTTAGACATCCGTTG
Baboon/1-1380      1022 GCCATGGATCATGGGGGATTTGCTGCCCACACTCTTAGGTGGCCTCTTAGACATCTGTTA
Rhesus/1-1376      1021 GCCATGGATCATGGGGGATTTGCTGCCCACACTCTTAGGTGGCCTCTTAGACATCTGTTA
Tarsier/1-1346      974 ACCATGCACAATGGGGAACTCACTAGCCACACTCCTAGGCCGCCTCCT---CATCCACTG
Marmoset/1-1351     974 GCCATGGATCATGGGGG-TTTGCTGCGCACACTCCTAGGCAGCCTCCGAGACATCCGTTG
Squirr mky/1-1332   982 GCAATGGATCGTGGGG-ATTTGCTGCCCACACTCCTAGGCAGCCTCTGAGACATCCATTG


Orangutan/1-1461   1160 GTGCCTAACCC-AAGCATCAGTTTGGCAGAGGCTGAGTCCCTCCTCTGTACTAGATACCA
Gorilla/1-1349     1049 GTGCCTAACCC-AAGCATCAGTTTGGCAGAGGCTGAGTCCCTCCTCTGTACTGGATACCA
Chimpanzee/1-1404  1104 GTGCCTAACCC-AAGCATCAGTTTGGCAGAGGCTGAGTCCCTCCTCTGTACTGGATACCA
Human/1-1431       1131 GTGCCTAACCC-AAGCATCAGTTTGGCAGAGGCCGAGTCCCTCCTCTGTACTGGATACCA
Gibbon/1-1400      1100 GTGCCTAACCC-AAGCATCAGTTTGGCAGAGGCTGAGTCCCTCCTCTGTATTGGATACCA
Baboon/1-1380      1082 GTGCCTAACCC-AAGCATCAGTTTGACAGAGGCTGAGTCCCTCCTCTGTACTGGGTACCA
Rhesus/1-1376      1081 GTGCCTAACCC-AAGCATCAGTTTGACAGAGGCTGAGTCCCTCCTCTGTACTGGGTACCA
Tarsier/1-1346     1031 GTGCCTTTCCCCAAGCATCAGTTTGGCAGTGGTTGGGTCCCTCTTCTGCACCAGGTACCT
Marmoset/1-1351    1033 GTACCTAATCC-AAGCATCAGTTTGGCAGAGGCTG--TCCCTCCTCTGTACTGGGTACCA
Squirr mky/1-1332  1041 GTGCCTAACCC-AAGCATCAGTTTGGCAGAGGCTG--TCCCTCCTCTGTACTGGGTACCA


Orangutan/1-1461   1219 GGTCAGCTTCCGTAGGGATGGGGAGATACCTGGCCCAGGGAGGAGATGAGGAGCAGCCCG
Gorilla/1-1349     1108 AGTCAGCTTCCGTAGGGATGGGGAGACACCTGGCCCAGGGAGGAGATGAGAAGCAGCCGG
Chimpanzee/1-1404  1163 CATCAGCTTCCGTAGGGATGGGGAGACACCTGGCCCAGCGAGGAGATGAGAAGCAGCCCG
Human/1-1431       1190 AGTCAGCTTCCATAGGGATGGGGAGACACCTGGCCCAGGGAGGAGATGAGAAGCAGCCCG
Gibbon/1-1400      1159 AGTCAGCTTCCGTAGGGATGGGGAGACACCTGGCCCAGGGAGGAGATGAGGAGCAGCCCG
Baboon/1-1380      1141 AGTCAGCTTCTGTAGGGATGGGGAGACACCTGGCCCAGGGAGGAGATGAGGAGCAGCCCG
Rhesus/1-1376      1140 AGTCAGCTTCTGTAGGGATGGGGAGACACCTGGCCCAGGGAG---ATGAGGAGCAGCCCG
Tarsier/1-1346     1091 AGTCAACTTCTATAGTGATGGGGAGACACTTGGCCCAGAAAG-ACA--AGAAGCAGACCA
Marmoset/1-1351    1090 AGTCAGCTTCTGTAGGGATGGGGAGACACGTGGCACAGAGAGGAGATGAGGAGCAGGCCA
Squirr mky/1-1332  1098 GGTCAGCTTCTGTAGGGATGGGGAGACACCTGGCACAGGAAGGAGATGAGGAGCAGGCCG


Orangutan/1-1461   1279 GATGGTGCTACA---TATGTCAGAGAGCAGGGCAGGAAGGGATCAGTGTGGATGCCAATG
Gorilla/1-1349     1168 GATGGTGCTACA---TATGTCAGAGAGCAGGGCAGGAAGGGATCAGTGTGGATGCCAATG
Chimpanzee/1-1404  1223 GATGGTGCTACA---TATGTCAGAGAGCAGGGCAGGAAGGGATCAGTGTGGCTGCCAATG
Human/1-1431       1250 GATGGTGCTACA---TATGTCAGAGAGCAGGGCAGGAAGGGATCAGTGTGGCTGCCAATG
Gibbon/1-1400      1219 GATGGTGCTACG---TATGTCAGAGAGCAGGGCAGGAAGGGATCAGTGTGGATGCCAGTG
Baboon/1-1380      1201 GATGGTGCTACA---TATGTCAGAGAGCAGGGCAGGAAGGGATCAGTGTGGATGCCAATG
Rhesus/1-1376      1197 GATGGTGCCACT---TATGTCAGAGAGGAGGGCAGGAAGGGATCAGTGTGGATGCCAATG
Tarsier/1-1346     1148 G---GTGGTAATGGGTATGTGAGGGTGCAGGGCAGGGAGGGGTCGGTGTGGATGCAGATG
Marmoset/1-1351    1150 G---GTGCTACA---TATGTCAGAGAGCAGGGCAGTAAGGGATCAGTGTGGATGCCAGTG
Squirr mky/1-1332  1158 G---GTGCTACA---TATGTCAGAGAGCAGGGCAGGAAGGGATCAG-GTGGATGCCAGTG
```

```
Orangutan/1-1461  1336 GTCAGGAGGGCGCCATGGAGTGAA---------------------------CCATGACCC
Gorilla/1-1349    1225 GTCAGGAGGGTGCCATGGAGTGAA---------------------------CCATGGCCC
Chimpanzee/1-1404 1280 GTCAGGAGGGCGCCATGGAGTGAA---------------------------CCATGGCCC
Human/1-1431      1307 GTCAGGAGGGCGCCATGGAGTGAA---------------------------CCATGGCCC
Gibbon/1-1400     1276 GTCAGGAGGGTGCCATGGAGTGAA---------------------------CCATGGCCC
Baboon/1-1380     1258 GTCAGGAGGGCGCCATGGGGTGAA---------------------------CCATGGCCC
Rhesus/1-1376     1254 GTCAGGAGGGCGCCATGGGGTGAA---------------------------CCATGGCCC
Tarsier/1-1346    1205 GTCAGGAAGACACCCTGAGGAGAAAGAACCATGACTCCCAGCAAGTAACTCCCCTCACCC
Marmoset/1-1351   1204 GTCAGGAGGGCGCCG-GGGGTGAA---------------------------CCATGACCC
Squirr mky/1-1332 1211 GTCAGGAGGGTGCTGTGGGGTGAA---------------------------CCAGAGGCC


Orangutan/1-1461  1369 CTGCCT-CCTCGCCAGCAGCTCCTCTGGGGAGGATACTCAAGACCCCCGGTCTCTCTCCT
Gorilla/1-1349    1258 CTGCCT-CCTCACCAGCAGCTCCTCTGGGGCTGATACTCAAGATCCCCCGTCTCTCTCCT
Chimpanzee/1-1404 1313 CTGCCT-CCTCACCAGCAGCTCCTCTGGGGCTGATACTCAAGACCCCCCGTCTCTCTCCT
Human/1-1431      1340 CTGCCT-CCTCACCAGCAGCTCCTCTGGAGCTGATACTCAAGACCCCCCGTCTCTCTCCT
Gibbon/1-1400     1309 CTGCCT-CCTCACCAGCAGCTCCTCTGGGGCTGATACTCAAGACCCCCCGTCTCTCTCCT
Baboon/1-1380     1291 CTGCCT-CCTCATCAGCAGCTCCTCTGGGGCTGATACTCAAGATCCC--GTCTCTCTCCT
Rhesus/1-1376     1287 CTGCCT-CCTCATCAGCAGCTCCTCTGGGGCTGATACTCAAGATCCC--GTCTCTCTCCT
Tarsier/1-1346    1265 CTGCCTACCCCACCAGTGGTTTCTCCGGGGCTGGTTCACAAGACCCC--ACCTCTCTCCT
Marmoset/1-1351   1236 CTGCCC-CCTCACCAGCAGCTCCTCTGGAGCTGAAACTCAAGACCCT--GTCTCTCTCCT
Squirr mky/1-1332 1244 GTGCC--CCTCACGAGCAGCTCCTCTGGGGCTGATACTCAAGACCCC--GTCTCTCTCCT


Orangutan/1-1461  1428 CACCCTCCTCTACCGGCTGC--CTCGGCTG------------------------GCTCAG
Gorilla/1-1349    1317 CACCCTCCTCT-CCCGCTGC--CTCGGCTG------------------------GCTCAG
Chimpanzee/1-1404 1372 CACCCTCCTCT-CCCGCTGC--CTCGGCTG------------------------GCTCAG
Human/1-1431      1399 CACCCTCCTCT-CCCGCTGC--CTCGGCTG------------------------GCTCAG
Gibbon/1-1400     1368 CAACCTCTTCT-CCCGCTGC--CTCGGCGG------------------------GCTCAG
Baboon/1-1380     1348 CACCCTCCTCT-CTCGCTGC--CTCGGCTG------------------------GCTCAG
Rhesus/1-1376     1344 CACCCTCCTCT-CTCGCTGC--CTCGGCTG------------------------GCTCAG
Tarsier/1-1346    1323 CT----------CCCCCTGC--CTCGGCTG------------------------GCTCAG
Marmoset/1-1351   1293 CTGCCTCCTCT-CCCGCTGCGGCTCAGGTGTTCGATGCCCGGGACTGCAGGTCTGCCCAG
Squirr mky/1-1332 1300 CTGCCTCCTCT-CCGGCTGC--CTCGGCTG------------------------GCTCAG
```
